# Burden of hepatitis B virus-associated liver cancer in Asia: findings from the global burden of disease study

**DOI:** 10.3389/fpubh.2026.1805052

**Published:** 2026-04-23

**Authors:** Xin-Jing Yang, Xue-Qi Yang, Xuan-Ying Mo, Jing Zuo, Yu-Chen Fan

**Affiliations:** 1 Department of Hepatology, Qilu Hospital of Shandong University, Jinan, China; 2 Hepatology Institute of Shandong University, Jinan, China

**Keywords:** Asia, global burden of disease, hepatitis B, Joinpoint model, liver cancer

## Abstract

**Background:**

Hepatitis B virus (HBV) infection is widespread in Asia. Of the many chronic diseases linked to HBV, among the most important is liver cancer. This study analysed the disease burden of chronic hepatitis B-associated liver cancer in Asia from 1990 to 2021.

**Methods:**

Data were obtained from the 2021 Global Burden of Disease study. We analyzed incidence, prevalence, deaths and disability-adjusted life years (DALYs) in Asian regions and countries from 1990 to 2021 by age and sex. Data processing and graph construction were performed using RStudio and BioWinford. The Joinpoint regression model was used to analyse the temporal data trends.

**Results:**

From 1990 to 2021, the disease burden of hepatitis B-related liver cancer in Asia decreased. A decline was observed in the rates of incidence (AAPC: −0.6), prevalence (AAPC: −0.1), deaths (AAPC: −1.0), and DALYs (AAPC: −1.2), even though their absolute numbers all increased. The number of incidence, prevalence, deaths and DALYs is highest among the middle-aged and older adults, and the highest rate is found among the older adults. Both the number and rate are highest in East Asia. Disease burdens differ by country.

**Discussion:**

The burden of hepatitis B-associated liver cancer in Asia has decreased over the past three decades, but remains non-negligible. Asian countries need to take corresponding measures against hepatitis B.

## Introduction

Liver cancer represents a major component of the disease burden attributable to hepatitis B virus infection (1). Among the liver diseases resulting from persistent hepatitis B infection, liver cancer is a particularly significant consequence **(**2, 3). Its pathogenesis, progression, and prognosis are closely linked to HBV-induced hepatic inflammation (4–7).

Previous studies have analyzed the global burden of hepatitis B and liver cancer separately. In recent years, the incidence, prevalence, mortality and disability-adjusted life years of hepatitis B infection in all age groups have been effectively controlled, but the number of them continues to increase (8). A similar situation occurs in the global disease burden of liver cancer (9). Since hepatitis B is a non-negligible cause of liver cancer, liver cancer caused by hepatitis B is still a great threat worldwide.

Among global regions, Asia bears a disproportionate burden of hepatitis infections, with half of the countries exhibiting the highest global burdens of hepatitis being Asian (10). Hepatitis B is a particularly critical subtype due to its high rate of chronic infection, strong causal association with liver cancer, and challenges in treatment. Across Asian countries with diverse income levels, approximately 180 million individuals are positive for hepatitis B surface antigen (11). Liver cancer caused by hepatitis B is therefore a major threat in Asia (12).

Vaccines, diagnostic tests, and antiviral therapies have been rigorously studied and widely implemented to mitigate the significant harm caused by hepatitis B. The central aim of such treatment is to prevent the development of liver cancer by arresting disease progression, thus improving survival and quality of life (4). However, curative treatment for chronic hepatitis B infection remains elusive (**13****)**. In 2016, the World Health Assembly adopted a resolution aiming for the elimination of viral hepatitis by 2030 (14). To contribute to this goal, the present study analysed the disease burden of chronic hepatitis B-associated liver cancer in Asia from 1990 to 2021.

## Materials and methods

### Data source

All data were obtained from the Global Burden of Diseases, Injuries, and Risk Factors Study (GBD) 2021^1^ (15). GBD 2021 provides comprehensive estimates of disease burden across time, age, sex, region and social factors, evaluated from the perspective of incidence, prevalence, deaths and disability-adjusted life years (DALYs). DALYs, introduced as a disease burden indicator in the World Bank’s 1993 World Development Report, are now endorsed by the WHO and the UN. GBD 2021 encompasses 371 diseases and injuries in 204 countries and regions.

Notably, the most recent GBD database (GBD 2023) does not include ‘Asia’ in the regional category. Using it would result in our data collection being insufficiently comprehensive and reliable. Therefore, the present study, based on GBD 2021, remains the most up-to-date research in the field of liver cancer caused by hepatitis B specifically in Asia. All the data in our research are derived from the GBD 2021 database, which provides a more comprehensive and intuitive collection of data for the Asian region.

In our research, we designed to utilize certain models to visualize the data in the GBD database into clear charts, so as to analyze the changing trends of the data with respect to different variables. The annual crude values and age-standardized estimates of the burden of liver cancer due to hepatitis B from the GBD database for the period from 1990 to 2021 in Asia were extracted. We investigated the number and age-standardized rate of incidence, prevalence, deaths and DALYs of the disease, considering the variables of time, age, sex, region, nation and socio-demographic index.

### Statistical analysis

The GBD provides age-standardized rates to facilitate valid comparisons across populations with different demographic structures using a uniform standard population. We calculated age-standardized incidence, deaths, prevalence, and DALYs rates for Asian populations using the global standard population provided by GBD. 95% uncertainty intervals (UIs) of these data were generated based on the 2.5th and 97.5th percentiles of 1,000 ordered draws. All rates are reported per 100,000 population.

Data processing, including table and figure construction, mainly utilized RStudio, with BioWinford also employed for image drawing. Joinpoint regression was used to analyse temporal trends and calculate annual percentage change (APC), reflecting the trends within specific periods, and average APC (AAPC), a comprehensive assessment of trends throughout the study period, calculated by linear regression. This model divides the research time range into different intervals through several connection points, and conducts trend fitting independently for each interval. Thus, it can objectively identify the turning points and their positions where significant changes occur in the time series. We can use it to observe the trend of data changes over time and identify the key connection points. Considering the trend of data changes over time, the final selected model in this study is 5 Joinpoints. The age-standardized indicator is considered on an upward trend if the corresponding APC and AAPC value is >0, and is considered on a downward trend if it is <0. We analysed the incidence, prevalence, deaths and DALYs of hepatitis B-related liver cancer across Asian regions and nations by age, sex, region, country and socio-demographic index (SDI). Sex classification was based on the GBD standard. The SDI, a composite indicator of socioeconomic development, incorporates total fertility rate, average education level and per-capita income. The larger the value, the higher the development status of the country.

### Nations and regions

Asian countries encompass Afghanistan, Armenia, Azerbaijan, Bahrain, Bangladesh, Bhutan, Brunei, Cambodia, China, Cyprus, Democratic People’s Republic of Korea, Georgia, India, Indonesia, Iran, Iraq, Israel, Japan, Jordan, Kazakhstan, Kuwait, Kyrgyzstan, Laos, Lebanon, Malaysia, Maldives, Mongolia, Myanmar, Nepal, Oman, Pakistan, Palestine, Philippines, Qatar, Republic of Korea, Saudi Arabia, Singapore, Sri Lanka, Syria, Tajikistan, Thailand, Timor-Leste, Turkey, Turkmenistan, United Arab Emirates, Uzbekistan, Vietnam and Yemen. Among the regions available in GBD 2021, we selected Central Asia, East Asia, South Asia, Southeast Asia, high income Asia-Pacific (including Brunei, Republic of Korea, Japan and Singapore), and North Africa and Middle East. These six regions collectively encompass the majority of the Asian continent and adjacent areas.

## Results

### Temporal trends

From 1990 to 2021, the overall number of patients with hepatitis B-caused liver cancer increased. However, the age-standardized rates of incidence, prevalence, deaths and DALYs all exhibited varying degrees of decline (Table 1).

**Table 1:** Age-standardized rates (per 100,000) and absolute numbers (in thousands) for incidence, prevalence, deaths and DALYs of liver cancer due to hepatitis B in Asia from 1990 to 2021.

Measures	Age-standardized rates			Number		
	1990	2021	AAPC -95 CI	1990	2021	AAPC -95 CI
Incidence	3.97 (3.42–4.55)	3.32 (2.71–4.08)	−0.6 (−0.6 to −0.5)	92,196.03 (79,883.60–105,984.20)	173,173.22 (140,487.27–213,578.56)	2.1 (2.0 to 2.1)
Prevalence	4.84 (4.21–5.54)	4.64 (3.79–5.68)	−0.1 (−0.2 to −0.1)	117,624.43 (102,049.20–134,614.83)	243,634.17 (198,369.84–299,526.06)	2.4 (2.3 to 2.4)
Deaths	3.92 (3.37–4.50)	2.89 (2.36–3.55)	−1.0 (−1.0 to −0.9)	89,378.44 (77,335.97–102,679.00)	149,742.77 (121,986.23–184,485.71)	1.7 (1.6 to 1.8)
DALYs	128.96 (111.69–148.25)	88.52 (72.58–109.06)	−1.2 (−1.3 to −1.1	3,185,840.68 (2,759,308.28–3,654,331.49)	4,669,732.10 (3,826,960.87–5,773,574.20)	1.3 (1.2 to 1.3)

AAPC, average annual percentage change; DALYs, disability-adjusted life years.

**Figure 1:**
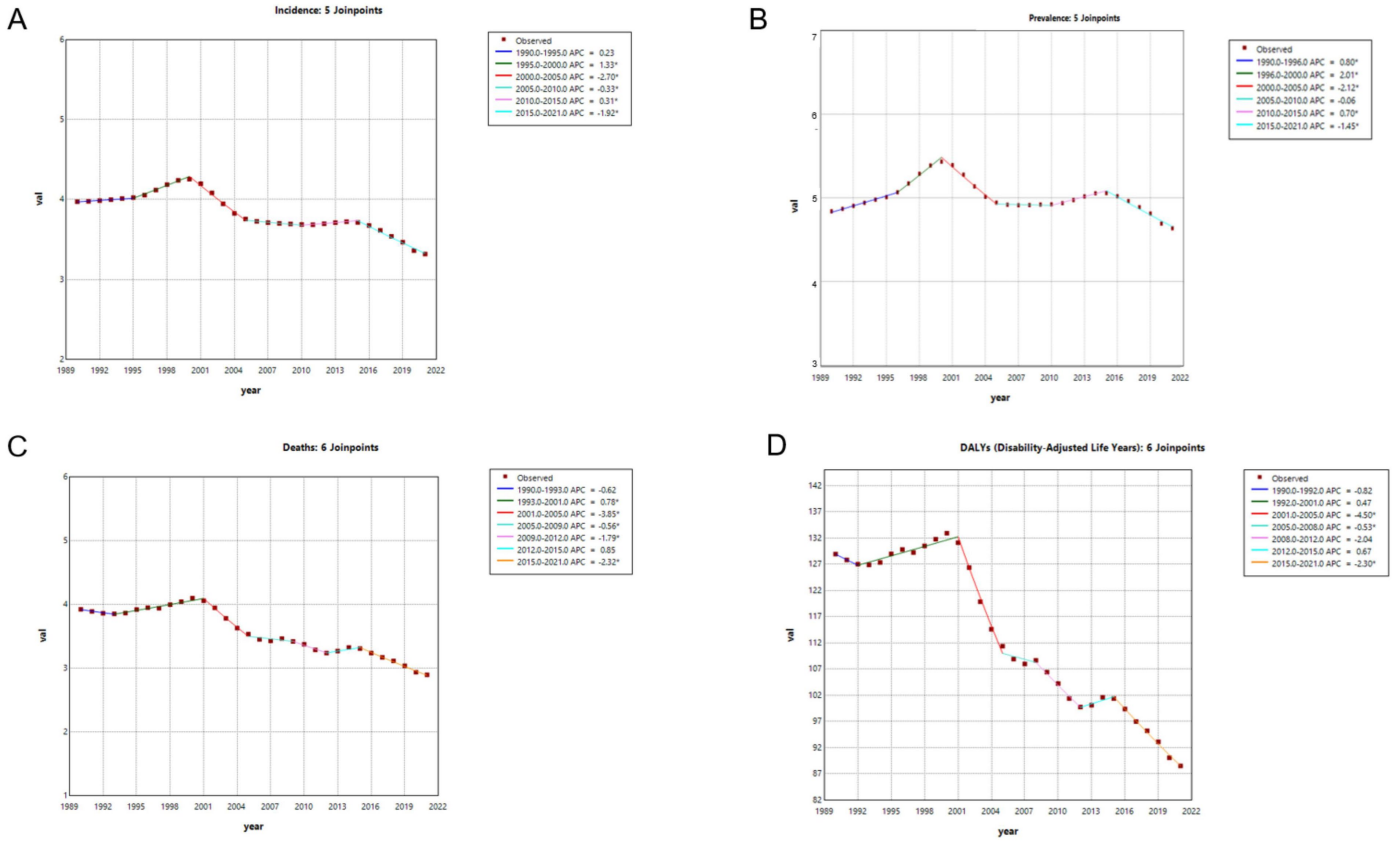
Temporal trends of the incidence **(A)**, prevalence **(B)**, deaths **(C)**, and DALYs **(D)** of HBV-associated liver cancer in Asia from 1990 to 2021. APC, annual percentage change; DALYs, disability-adjusted life years; HBV, hepatitis B virus. *Indicates that the annual percent change (APC) is significantly different from zero at the alpha = 0.05 level. Final selected model: 5 Joinpoints.

The reduction in the age-standardized DALYs rate was most pronounced, with an AAPC of −1.2%, corresponding to a decrease from 128.96 (95% UI: 111.69–148.25) per 100,000 in 1990 to 88.52 (95% UI: 72.58–109.06) per 100,000 in 2021. The temporal trend curve demonstrates a significant overall decline in the DALYs rate of hepatitis B-associated liver cancer from 1990 to 2021. Joinpoint regression identified significant changes in the DALYs trend in 1992, 2001, 2005, 2008, 2012, and 2015. A slight increase in the DALYs rate was observed between 1992 and 2001, followed by a sharp decline. Despite some fluctuations thereafter, the overall trend remained significantly downward through 2021, with the steepest decline occurring between 2001 and 2005 (APC = −4.50%) (Figure 1D, and Supplementary Table 1).

Disease incidence, prevalence and deaths changed much more slowly than DALYs (Figures 1A–C). They showed the same overall downward trajectory as DALYs from 1990 to 2021, with occasional concurrent increases during similar periods of time.

### By age and sex

In the analysis of the disease burden of hepatitis B-associated liver cancer by age and sex, the most notable feature is the significant sex disparity in the incidence rate, prevalence rate, death rate and DALYs. This disparity persists even after accounting for age-specific rates.

**Figure 2:**
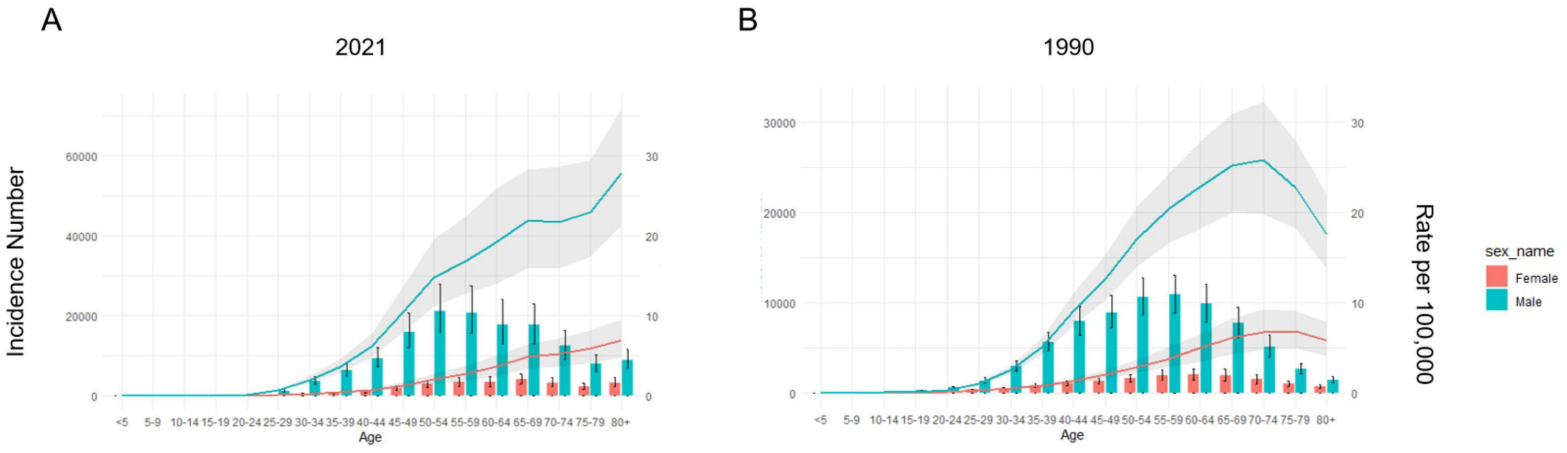
Incidence number and rate of hepatitis B-related liver cancer for men and women changed by age in Asia in 2021 **(A)** and 1990 **(B)**.

The population was stratified into 17 five-year age groups. The overall pattern showed an initial increase in case numbers followed by a decrease, although a slight increase was noted in individuals aged over 80 years. Case numbers were negligible in those under 25 years. The burden was concentrated in middle-aged and aged populations, with the highest number of cases observed in the 50–54 age group in 2021 [males: 21,236.90 (95% UI: 16,083.98–28,156.37); females: 2,958.05 (95% UI: 2,187.75–3,922.93)], where males outnumbered females by approximately sevenfold. Across the total population, male cases (76,625.79; 95% UI: 64,579.53–89,845.32) significantly outnumbered female cases (15,570.24; 95% UI: 12,643.28–18,690.85). The peak age group for case numbers differed by sex: 50–54 years for males (see above) and 65–69 years for females (4,190.09; 95% UI: 2,951.89–5,614.13). Incidence rates generally increased with age, peaking in the 80 + years group, followed by the 75–79 years group. In the 80 + age group, the rate per 100,000 was 27.84 (95% UI: 21.28–36.00) for males and 6.97 (95% UI: 4.86–9.58) for females, representing an approximately fourfold higher rate in males (Figure 2A and Supplementary Table 2).

In 1990, the incidence number of hepatitis B-associated liver cancer was generally lower than but followed a similar age distribution to that in 2021. However, the incidence rate was higher in 1990 than in 2021. In 1990, the age groups with the highest incidence rate were 70–74 years (25.76; 95% UI: 19.79–32.25) for men and 75–79 years (6.89; 95% UI: 5.00–9.11) for women (Figures 2B and Supplementary Table 2).

Age-specific trends of disease prevalence, deaths and DALYs rates for both sexes by age largely paralleled those of the incidence rate. Unlike the other measures, the DALYs rate exhibited an initial increase followed by a decrease. The age group with the highest rate for DALYs in 1990 was 55–59 years (783.92; 95% UI: 630.75–950.63), shifting to 65–69 years (602.05; 95% UI: 439.46–786.45) (Supplementary Figure 1, Supplementary Table 2).

### By region

**Figure 3:**
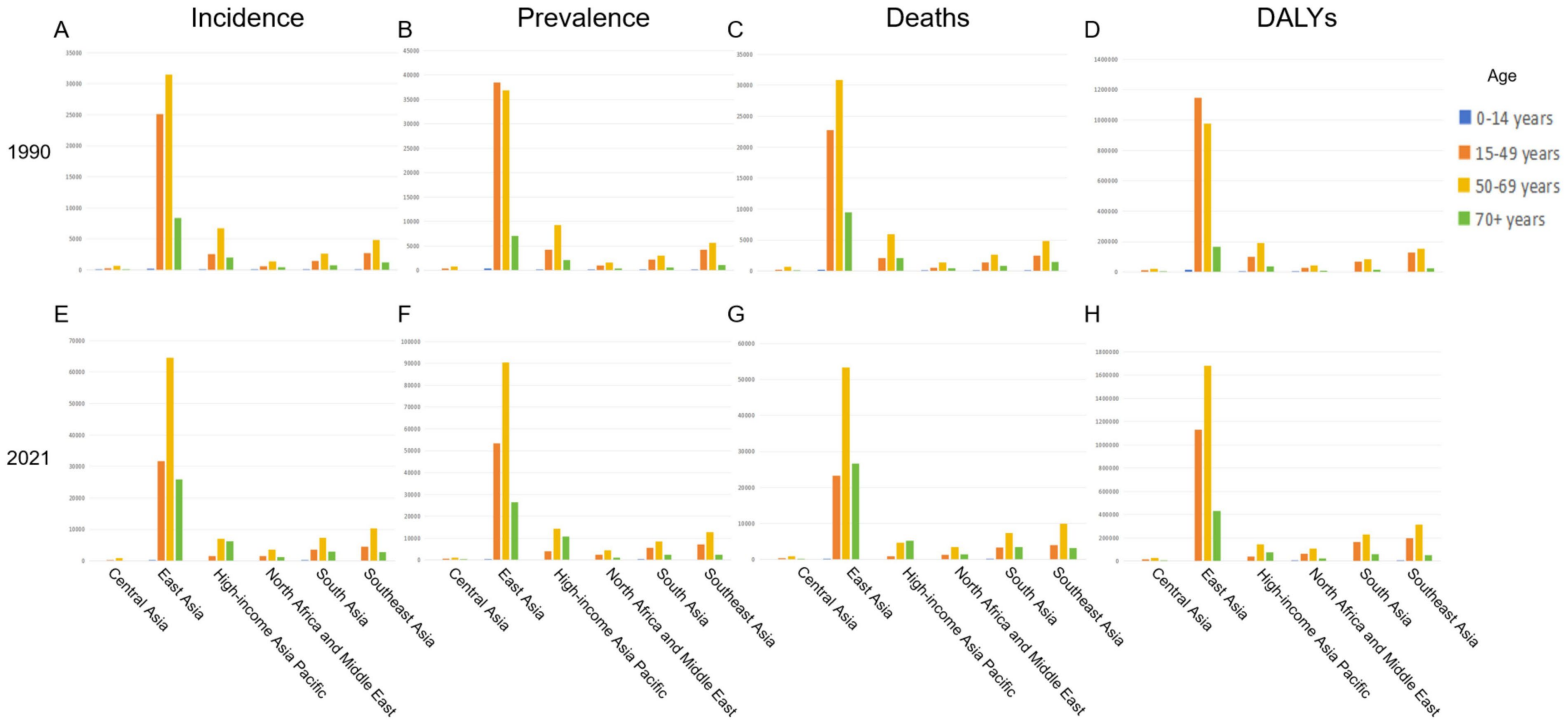
Incidence, deaths, and DALYs of hepatitis B-related liver cancer among different age groups in various regions of Asia in 1990 and 2021. **(A)** Incidence rate of HBV-related liver cancer among different age groups in various regions of Asia in 1990. **(B)** Prevalence rate of HBV-related liver cancer among different age groups in various regions of Asia in 1990. **(C)** Deaths rate of HBV-related liver cancer among different age groups in various regions of Asia in 1990. **(D)** DALYs rate of HBV-related liver cancer among different age groups in various regions of Asia in 1990. **(E)** Incidence rate of HBV-related liver cancer among different age groups in various regions of Asia in 2021. **(F)** Prevalence rate of HBV-related liver cancer among different age groups in various regions of Asia in 2021. **(G)** Deaths rate of HBV-related liver cancer among different age groups in various regions of Asia in 2021. **(H)** DALYs rate of HBV-related liver cancer among different age groups in various regions of Asia in 2021. DALYs, disability-adjusted life years; HBV, hepatitis B virus.

Among the six regions, East Asia had the highest disease burden, with its number of incidence, prevalence, deaths and DALYs of hepatitis B-associated liver cancer being the highest in both 1990 and 2021. These figures were substantially lower in Southeast Asia, South Asia, High-income Asia Pacific, and North Africa and the Middle East, and lowest in Central Asia. In East Asia, the age group with the highest number of disease incidences and deaths was 50–69 years old, with the figures being 31465.04 (95% UI: 25574.06–38091.54)and 30866.01 (95% UI: 25080.19–37228.86) respectively in 1990. The number of prevalence and DALYs in the 15–49 age group was higher than those in the 50–69 age group. Among them, the prevalence number aged 15–49 reached 38483.88 (95% UI: 31963.97–46071.82), and the number of DALYs reached 1147442.58 (95% UI: 952550.35–1371699.54). In all regions except East Asia, the highest numbers of incidence, prevalence, deaths and DALYs occurred in the age groups 50–69. Number of incidence, prevalence, mortality, and DALYs were also relatively high in the 15–49 years age group in East Asia, while they were relatively low in the 70 + years age group. The number and rate of people under 15 affected by the disease burden were even negligible. Notably, in East Asia, which had the most severe disease burden, the number of incidence, prevalence, deaths and DALYs in the 70 + age groups were significantly lower than in the 15–49 age group. However, in other regions, the gap between the data of these two age groups is not as large. (Figures 3A–D and Supplementary Table 3).

In 2021, the number of cases increased in all the six regions, and the overall situation was similar to that of 1990. East Asia remained the region with the highest disease burden, and the 50–69 years age group continued to bear the greatest burden. However, unlike in 1990, the number of prevalence and DALYs for the 50–69 age group in East Asia exceeded that of the 15–49 age group in 2021, with numbers of 90317.33 (95% UI: 67849.44–119632.98) and 1679082.63 (95% UI: 1264449.64–2225351.02) separately. A reversal from 1990, in East Asia, the number of deaths among those over 70 slightly exceeded that of the 15–49 age groups in 2021 (Figures 3E–H and Supplementary Table 3).

Due to differences in the population base, the disparities in rates of disease incidence, prevalence, deaths and DALYs among regions were less pronounced than that in numbers. While East Asia remained the region with the highest burden. In East Asia, the group with the highest incidence rate was 70 + years, in both 1990 (21.42; 95% UI: 17.52–26.25) and 2021 (20.95; 95% UI: 15.99–27.02) (Supplementary Table 3). The rate of incidence, prevalence, deaths and DALYs in most regions decreased in 2021 compared to 1990. However, in South Asia and North Africa and Middle East, these rates increased in this period.

### By nation

**Figure 4:**
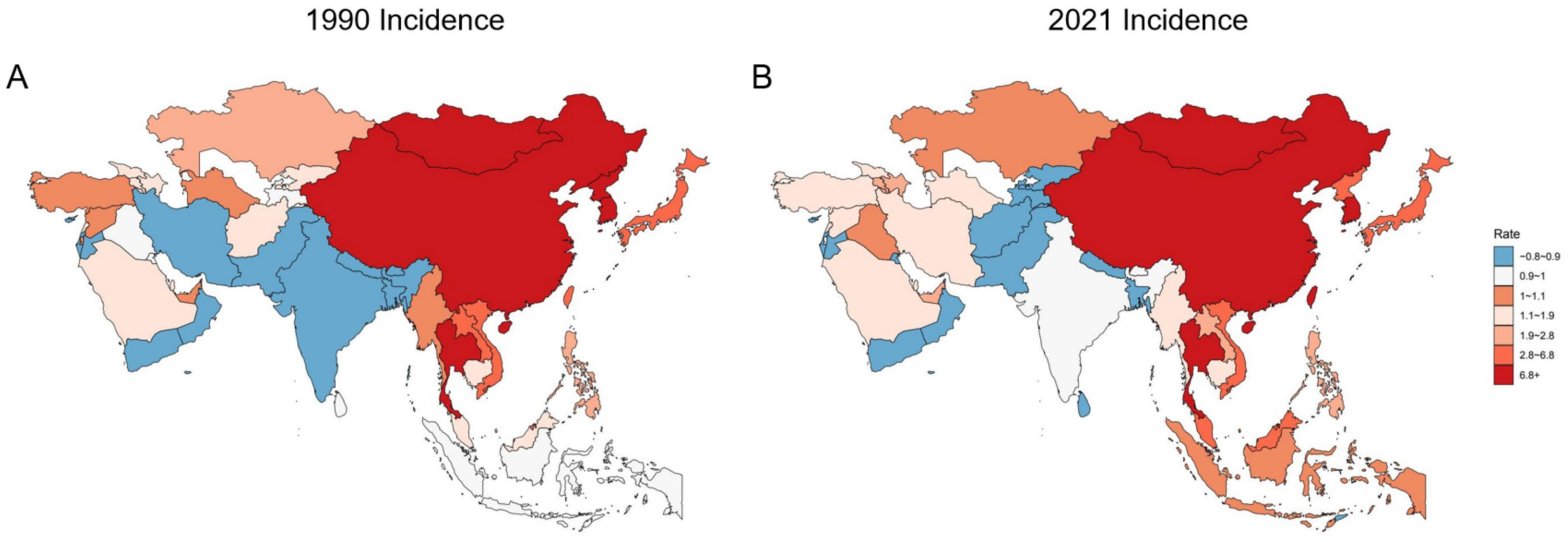
Incidence rates of liver cancer due to hepatitis B in Asian countries in 1990 **(A)** and 2021 **(B)**.

In 1990, the Republic of Korea had the highest age-standardized incidence rate of hepatitis B-associated liver cancer in Asia (22.19 per 100,000; 95% UI: 15.78–28.39), followed by Mongolia (17.56; 95% UI: 11.40–26.41). Both countries had rates substantially higher than third-ranked Brunei (7.05; 95% UI: 4.84–9.96). The lowest incidence rate was observed in Israel (0.36; 95% UI: 0.25–0.51), with the rate in the Republic of Korea being nearly 62 times higher. By 2021, incidence rates had generally declined across Asia, with only a few countries showing increases. Mongolia had the highest incidence rate in 2021 (16.61; 95% UI: 10.77–24.42), followed by the Republic of Korea (11.18; 95% UI: 8.91–14.02) and China (5.73; 95% UI: 4.48–7.38). Kuwait had the lowest incidence rate (0.33; 95% UI: 0.23–0.47) (Table 2 and Figure 4).

**Table 2:** Age-standardized rates (per 100,000) for incidence of liver cancer due to hepatitis B in Asian countries in 1990 and 2021.

Country	Incidence (95% UI)	
	1990	2021
Republic of Korea	(22.19; 15.78–28.39)	(11.18; 8.91–14.02)
Mongolia	(17.56; 11.4–26.41)	(16.61; 10.77–24.42)
Brunei	(7.05; 4.84–9.96)	(4.32; 3.04–5.99)
Vietnam	(6.96; 4.53–9.71)	(5.25; 3.40–7.94)
China	(6.58; 5.45–7.84)	(5.73; 4.48–7.38)
Thailand	(6.41; 4.69–8.69)	(4.35; 2.97–6.18)
Democratic People’s Republic of Korea	(6.23; 3.35–9.46)	(4.03; 2.52–5.98)
Singapore	(5.77; 4.84–6.80)	(4.37; 3.50–5.29)
Laos	(4.40; 2.73–6.47)	(2.55; 1.63–3.86)
Taiwan (Province of China)	(4.36; 3.72–5.08)	(5.28; 4.24–6.51)
Qatar	(3.78; 2.45–5.58)	(4.17; 2.64–6.48)
Cambodia	(3.60; 1.80–6.88)	(2.22; 1.08–4.43)
Philippines	(3.27; 2.24–4.34)	(2.43; 1.96–3.03)
Maldives	(3.12; 2.15–4.37)	(1.71; 1.14–2.47)
United Arab Emirates	(3.10; 1.89–4.67)	(3.20; 2.08–4.79)
Saudi Arabia	(2.88; 1.78–4.55)	(1.98; 1.28–2.88)
Kazakhstan	(2.81; 1.97–3.82)	(1.01; 0.68–1.45)
Bahrain	(2.60; 1.71–3.70)	(1.35; 0.88–2.02)
Palestine	(2.32; 1.50–3.49)	(1.69; 1.19–2.41)
Malaysia	(2.31; 1.69–3.02)	(2.95; 2.13–4.01)
Syria	(2.30; 1.47–3.44)	(1.55; 0.95–2.36)
Japan	(2.16; 1.84–2.49)	(1.27; 1.04–1.52)
Afghanistan	(2.14; 1.34–3.29)	(2.02; 1.25–3.03)
Armenia	(1.85; 1.24–2.58)	(1.32; 0.87–1.87)
Kyrgyzstan	(1.74; 1.24–2.40)	(0.76; 0.49–1.11)
Azerbaijan	(1.62; 0.87–2.70)	(1.71; 0.85–3.17)
Kuwait	(1.56; 1.15–2.04)	(0.33; 0.23–0.47)
Lebanon	(1.52; 1.09–2.10)	(1.04; 0.73–1.42)
Turkmenistan	(1.51; 1.05–2.04)	(1.22; 0.76–1.89)
Iraq	(1.50; 1.00–2.13)	(1.51; 0.96–2.17)
Myanmar	(1.47; 0.68–2.86)	(1.21; 0.60–2.48)
Timor-Leste	(1.41; 0.88–2.28)	(1.10; 0.63–1.98)
Turkey	(1.39; 1.02–1.84)	(1.19; 0.86–1.62)
Georgia	(1.22; 0.89–1.68)	(0.93; 0.63–1.30)
Oman	(1.21; 0.71–2.01)	(1.23; 0.79–1.78)
Bhutan	(1.03; 0.54–1.63)	(1.05; 0.62–1.68)
Tajikistan	(1.01; 0.60–1.64)	(0.72; 0.37–1.24)
Iran	(0.99; 0.80–1.28)	(1.22; 1.06–1.41)
Yemen	(0.98; 0.35–2.00)	(0.59; 0.31–1.09)
Indonesia	(0.87; 0.58–1.32)	(1.03; 0.61–1.65)
Sri Lanka	(0.83; 0.61–1.06)	(0.53; 0.32–0.85)
India	(0.78; 0.64–0.93)	(0.95; 0.80–1.14)
Jordan	(0.76; 0.46–1.24)	(0.46; 0.29–0.67)
Bangladesh	(0.73; 0.52–1.04)	(0.63; 0.40–0.96)
Nepal	(0.51; 0.32–0.77)	(0.81; 0.48–1.24)
Cyprus	(0.49; 0.32–0.74)	(0.44; 0.28–0.69)
Pakistan	(0.41; 0.29–0.56)	(0.50; 0.36–0.68)
Israel	(0.36; 0.25–0.51)	(0.37; 0.25–0.53)

The burdens of prevalence, deaths and DALYs in Asian countries generally mirrored those of incidence patterns. The notable difference is that in 2021, the prevalence rate in the Republic of Korea remained the highest, which might be related to the fact that the effectiveness of the country’s prevention measures takes time to be realized. Detailed data are available in Supplementary Table 4 and Supplementary Figure 2.

### By SDI

**Figure 5:**
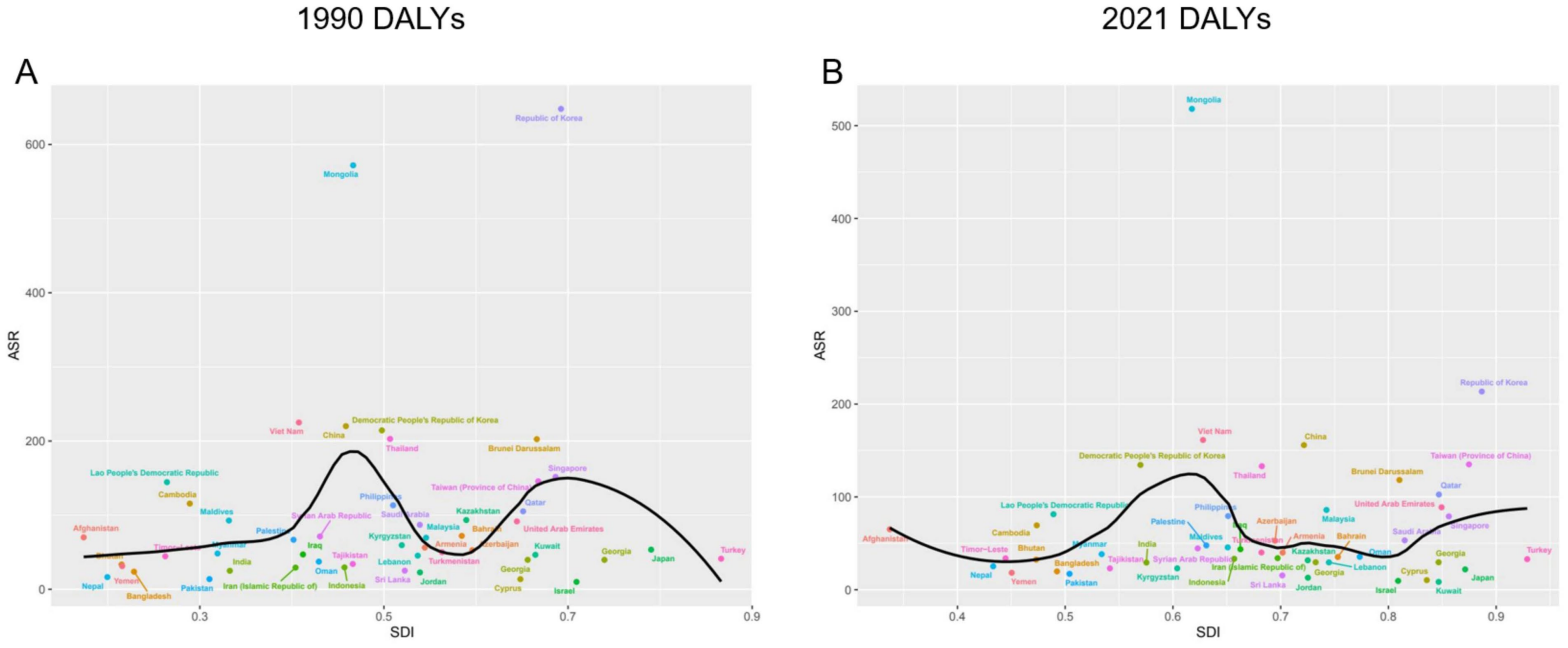
DALYs rate of HBV-related liver cancer in different Asian countries by SDI in 1990 **(A)** and 2021 **(B)**. ASR, age-standardized rate; DALYs, disability-adjusted life years; HBV, hepatitis B virus; SDI, socio-demographic index.

The SDI was not significantly correlated with DALYs rates attributable to hepatitis B-associated liver cancer in Asia. In 1990, countries with higher age-standardized DALYs rates were predominantly clustered in the medium-high SDI range, with two distinct peak segments observable. The Republic of Korea (medium-high SDI) and Mongolia (medium SDI) exhibited DALYs rates substantially higher than all other Asian countries (Figure 5A).

By 2021, the DALYs rate in the Republic of Korea had decreased markedly and was no longer an outlier, with the SDI of this country improved meanwhile. However, that of Mongolia still led by a wide margin. Unlike in 1990, countries with high SDI levels in 2021 demonstrated relatively high DALY rates among Asian nations (Figure 5B).

The incidence, prevalence and mortality of diseases also have no strong correlation with SDI, and their changing trends are highly similar to those of DALYs (Supplementary Figure 3).

## Discussion

This study provides a comprehensive analysis of the disease burden of hepatitis B-related liver cancer in Asia from 1990 to 2021. The burden of this cancer in Asia has fluctuated but decreased over the past 30 years. Its incidence, prevalence and death rates all increased during the 1990s, before declining again after 2000. Numerous studies have shown that many Asian countries have consistently placed great emphasis on the prevention and control of hepatitis B among all segments of the population. These measures include actively providing widespread vaccination against hepatitis B, especially for the mother-infant group, promoting more effective anti-hepatitis B virus drugs, and strengthening the prevention of liver cancer among high-risk groups (16–19). In the past three decades, the epidemiological patterns of both hepatitis B and liver cancer in Asia have undergone significant and ongoing changes (20–22). The trends revealed in this study can help practitioners to assess the effectiveness of current prevention efforts and plan resources rationally to further optimize policies.

This study revealed an extreme sex difference in the burden of hepatitis B-related liver cancer, with incidence, prevalence, death and DALYs rates all being significantly higher in men than in women. This substantial sex difference may be linked to several factors. First, it may be linked to physiological and immunological differences between the sexes. Second, behavioural factors play a crucial role, as men typically have greater exposure to high-risk factors such as excessive alcohol consumption and obesity (23, 24) Furthermore, differential access to preventive healthcare must be considered. Notably, hepatitis B treatment coverage varies between men and women, which may contribute to the disparity (25, 26). Studies have shown that women in Asia are less likely to receive hepatitis B treatment. Nonetheless, women show a significantly lower prevalence than men in our study, indicating that this factor has less influence on the burden of disease. In addition, from the results in our study, it can be inferred that the fact that male patients are facing a heavier disease burden might attract particular attention from the medical industry, which could lead to an increase in the treatment rate for male patients. Thus for the prevention and control of liver cancer caused by hepatitis B, the government should strive to ensure the fairness of medical conditions for different genders. At the same time, it should also focus on reducing the exposure of men to risk factors. Regarding age distribution, the older people was found to bear a heavier disease burden. Two age groups, 50–54 and 55–59, had the largest numbers of cases. In terms of incidence, in 1990, it was highest in the 70–74 age group, while in 2021, the highest incidence was in the 80 + age group. Although the absolute number of cases among older people was not the largest overall, their prevalence rate was extremely high. This might be related to the trend of population aging. Therefore, in the process of disease, it is necessary to not only control the aging of the population but also ensure the effective treatment of aged patients and prevent the increase of middle-aged cases.

Moreover, the disease burden varied by region and country. Within Asia, East Asia carried the heaviest burden of hepatitis B-related liver cancer. In contrast, the burden was lighter in South Asia, Southeast Asia, the high-income Asia-Pacific region, and the Middle East and North Africa, while Central Asia reported the lowest levels. At the country level, the Republic of Korea and Mongolia had the heaviest disease burdens. These geographical differences are closely linked to disparities in healthcare systems and public health measures. Hepatitis B vaccination coverage and the availability of antiviral treatments differ widely across Asia due to variations in medical infrastructure, resources and policy implementation (25, 27). Consequently, the impact of hepatitis B-related liver cancer, which is a largely preventable and manageable condition, shows great differences across Asia. Many countries in Asia are actively addressing hepatitis B and liver cancer, striving to control hepatitis B and seeking effective treatment drugs for liver cancer (28, 29). We found a significant decrease in incidence in the Republic of Korea. In 1990, the Republic of Korea had the highest incidence rate of hepatitis B-associated liver cancer among all Asian countries. However, the government and the private sector have since made continuous efforts to control hepatitis B and liver cancer to reduce the harm they cause (30, 31). Hepatitis B vaccination was introduced nationwide in 1995, and by 2021, the incidence rate had decreased substantially in the Republic of Korea (32). This indicates that the Republic of Korea’s measures to combat hepatitis B-related liver cancer have remarkable achievements, which serves as a model and can be emulated by Asian countries in terms of vaccine policies and treatment methods. However, we have observed that the disease burden of liver cancer caused by hepatitis B has increased in some regions and countries. These areas should follow the examples of those that have achieved success in disease prevention and control. Countries should enhance mutual exchanges to jointly promote the reduction of the disease’s harm in Asia.

This study also examined the association between the SDI and the burden of hepatitis B-related liver cancer across Asian countries from 1990 to 2021. The analysis indicated no clear linear correlation between a country’s overall SDI and its burden from this disease. One finding was that countries with a medium SDI consistently bore a relatively high burden in both 1990 and 2021, suggesting that factors beyond general development level play a critical role. Furthermore, the relationship between SDI and disease burden changed significantly over the three-decade study period. In 1990, the burden was more concentrated in medium-SDI countries. By 2021, while this group remained heavily affected, the distribution had shifted, with some high-SDI countries also showing greater burdens, possibly reflecting aging populations. Conversely, several low-to-medium SDI countries showed a great decrease in burden, likely linked to successful vaccination programs and improved antiviral therapy access. Therefore, regardless of whether a country has a high or low SDI, it should pay attention to the control of hepatitis B and liver cancer. Efforts should be made to ensure that the prevention and treatment of diseases progress along with the development of the country.

These findings hold significant implications for the advancement of public health strategies across Asia. Intervention strategies should differ according to age group. Of particular concern is the substantial disease burden carried by the older adults, which necessitates focused efforts on early detection, accessible treatment and management of complications within this group. Regarding sex, the management of male exposure to hepatitis B risk factors should be strengthened. This includes strengthening public health efforts to reduce male exposure to key risk factors such as alcohol and obesity, alongside ensuring equitable access to vaccination and screening services for all genders. Furthermore, the regional differences in disease burden emphasize the importance of region-specific solutions. At present, it is highly concerned about reducing the disease burden caused by hepatitis B and liver cancer globally (33–35). As an area severely affected by hepatitis B-related liver cancer, Asia should strengthen the prevention and control of these diseases even more. A number of countries have already begun to adopt such approaches, serving as valuable models for others (36–38). Our study can provide guidance for Asian regions and countries, helping them further implement hepatitis B control measures and liver cancer treatments that are carefully adapted to their unique conditions, including existing healthcare infrastructure, economic resources, cultural contexts and local epidemiological patterns.

This study has several limitations. In some countries, liver cancer screening may not be sufficiently comprehensive, potentially leading to inaccuracies in burden estimates. Reporting of hepatitis B cases, particularly in some developing countries, may also be incomplete, introducing data incomplete. These limitations can be related to the fact that we used only one database in this study. This indicates that further research requires the integration of varies databases to facilitate data collection. More comprehensive and accurate databases still need to be published. This study examined the disease burden of hepatitis B-related liver cancer but did not address other chronic liver diseases attributable to HBV, which need to be taken a deeper look in the future. Additionally, the burden among individuals with chronic HBV infection who have not yet progressed to advanced liver disease remains to be fully elucidated. More research on the correlation between hepatitis B and liver cancer needs to be carried out in the future.

## Conclusion

The findings show that although the disease burden fluctuated periodically, most metrics followed a downward trend. The disease largely concentrate on middle-aged and older adults, with males consistently outnumbering females. Regionally, East Asia carried the heaviest burden, with the Republic of Korea and Mongolia having the highest incidence and death rates. The study concludes that the development and implementation of tailored prevention and control strategies, which are designed in accordance with the distinct epidemiological patterns and healthcare situations of specific Asian regions, could contribute meaningfully to further reducing the public health impact of hepatitis B-associated liver cancer in the future. Specifically, there are notable differences across regions in terms of disease epidemics, population structure, access to medical resources, and the existing foundation for hepatitis B control. As a result, uniform strategies are often ineffective. Only by tailoring interventions to local conditions, such as optimizing vaccination strategies, strengthening screening for high-risk populations, and increasing the coverage of antiviral therapy, can we truly enhance prevention efficiency and further reduce the disease burden associated with hepatitis B-related liver cancer.

## Data Availability

The datasets presented in this study can be found in online repositories. The names of the repository/repositories and accession number(s) can be found in the article/Supplementary material.

## Footnotes

1
https://gbd2021.healthdata.org/gbd-results/
