# Supplementary material for: Burden of hepatitis B virus-associated liver cancer in Asia: findings from the global burden of disease study
Source: Front Public Health. 2026 Apr 23;14:1805052. doi: 10.3389/fpubh.2026.1805052 (PMC13149405; doi:10.3389/fpubh.2026.1805052)
Supplement: Supplementary file 1 [file Supplementary_File_1.docx]

**Supplementary Table 1: Temporal trend of incidence, prevalence, mortality and DALYs rate of HBV associated liver cancer in Asia from 1990 to 2021.**

| **Year** | **Incidence** | | **Prevalence** | | **Mortality** | | **DALYs** | |
| --- | --- | --- | --- | --- | --- | --- | --- | --- |
|  | **Rate per 100,000** | **SE** | **Rate per 100,000** | **SE** | **Rate per 100,000** | **SE** | **Rate per 100,000** | **SE** |
| **1990** | 3.97 | 0.29 | 4.84 | 0.34 | 3.86 | 0.23 | 128.96 | 9.33 |
| **1991** | 3.98 | 0.26 | 4.87 | 0.30 | 3.86 | 0.28 | 127.85 | 8.90 |
| **1992** | 3.99 | 0.24 | 4.90 | 0.28 | 3.92 | 0.29 | 127.04 | 8.85 |
| **1993** | 4.00 | 0.22 | 4.94 | 0.26 | 3.89 | 0.28 | 126.88 | 8.58 |
| **1994** | 4.01 | 0.22 | 4.98 | 0.27 | 3.94 | 0.24 | 127.38 | 7.62 |
| **1995** | 4.02 | 0.23 | 5.01 | 0.28 | 3.92 | 0.23 | 129.01 | 7.18 |
| **1996** | 4.06 | 0.22 | 5.07 | 0.26 | 4.00 | 0.24 | 129.82 | 7.15 |
| **1997** | 4.12 | 0.22 | 5.17 | 0.26 | 4.04 | 0.25 | 129.25 | 7.25 |
| **1998** | 4.19 | 0.23 | 5.29 | 0.26 | 4.10 | 0.25 | 130.52 | 7.57 |
| **1999** | 4.24 | 0.24 | 5.39 | 0.27 | 3.78 | 0.23 | 131.78 | 7.68 |
| **2000** | 4.25 | 0.25 | 5.43 | 0.29 | 4.06 | 0.25 | 132.93 | 7.32 |
| **2001** | 4.20 | 0.23 | 5.39 | 0.27 | 3.63 | 0.20 | 131.13 | 7.64 |
| **2002** | 4.08 | 0.21 | 5.28 | 0.26 | 3.53 | 0.19 | 126.39 | 7.46 |
| **2003** | 3.94 | 0.19 | 5.14 | 0.24 | 3.45 | 0.20 | 119.85 | 6.71 |
| **2004** | 3.82 | 0.19 | 5.01 | 0.24 | 3.43 | 0.20 | 114.62 | 5.58 |
| **2005** | 3.75 | 0.20 | 4.94 | 0.25 | 3.46 | 0.19 | 111.38 | 5.72 |
| **2006** | 3.73 | 0.19 | 4.92 | 0.24 | 3.42 | 0.20 | 108.91 | 5.80 |
| **2007** | 3.71 | 0.19 | 4.91 | 0.23 | 3.37 | 0.20 | 107.99 | 5.99 |
| **2008** | 3.70 | 0.19 | 4.92 | 0.23 | 3.29 | 0.20 | 108.68 | 5.53 |
| **2009** | 3.70 | 0.20 | 4.92 | 0.25 | 3.23 | 0.21 | 106.43 | 6.13 |
| **2010** | 3.69 | 0.22 | 4.92 | 0.27 | 3.27 | 0.20 | 104.24 | 5.93 |
| **2011** | 3.69 | 0.20 | 4.94 | 0.25 | 3.32 | 0.22 | 101.40 | 5.94 |
| **2012** | 3.70 | 0.20 | 4.97 | 0.25 | 3.30 | 0.24 | 99.75 | 6.00 |
| **2013** | 3.71 | 0.21 | 5.02 | 0.27 | 3.24 | 0.25 | 100.13 | 5.78 |
| **2014** | 3.72 | 0.23 | 5.05 | 0.30 | 3.17 | 0.27 | 101.59 | 6.34 |
| **2015** | 3.71 | 0.26 | 5.06 | 0.34 | 3.11 | 0.30 | 101.38 | 6.75 |
| **2016** | 3.67 | 0.25 | 5.02 | 0.33 | 3.04 | 0.31 | 99.38 | 7.50 |
| **2017** | 3.61 | 0.25 | 4.96 | 0.34 | 2.89 | 0.30 | 97.01 | 8.59 |
| **2018** | 3.54 | 0.29 | 4.89 | 0.41 | 3.95 | 0.24 | 95.24 | 8.90 |
| **2019** | 3.47 | 0.36 | 4.81 | 0.49 | 3.85 | 0.27 | 93.13 | 9.67 |
| **2020** | 3.36 | 0.33 | 4.69 | 0.47 | 3.95 | 0.24 | 90.09 | 9.04 |
| **2021** | 3.32 | 0.35 | 4.64 | 0.48 | 2.94 | 0.29 | 88.52 | 9.31 |

**Supplementary Table 2: Sex-specific incidence, deaths, prevalence and DALYs of HBV associated liver cancer in different age groups in Asia in 1990 and 2021.**

| **Measure** | **Age** | **1990Number (95% UI)** | | **2021Number (95% UI)** | | **1990Rate (95% UI), per 100,000 population** | | **2021Rate (95% UI), per 100,000 population** | |
| --- | --- | --- | --- | --- | --- | --- | --- | --- | --- |
|  |  | **Male** | **Female** | **Male** | **Female** | **Male** | **Female** | **Male** | **Female** |
| **Incidence (95% UI), per 100,000 population** | **<5** | ( 0 0,0) | ( 0 0,0) | ( 0 0,0) | ( 0 0,0) | ( 0 0,0) | ( 0 0,0) | ( 0 0,0) | ( 0 0,0) |
|  | **5 to 9** | ( 0 0,0) | ( 0 0,0) | ( 0 0,0) | ( 0 0,0) | ( 0 0,0) | ( 0 0,0) | ( 0 0,0) | ( 0 0,0) |
|  | **10 to 14** | ( 201.90 171.47,243.09) | ( 66.77 53.13,82.63) | ( 98.70 80.23,127.73) | ( 39.85 30.83,50.50) | ( 0.12 0.10,0.14) | ( 0.04 0.03,0.05) | ( 0.05 0.04,0.07) | ( 0.02 0.02,0.03) |
|  | **15 to 19** | ( 314.22 263.02,378.50) | ( 108.39 88.60,132.41) | ( 164.04 136.74,209.52) | ( 73.24 59.20,92.31) | ( 0.19 0.16,0.23) | ( 0.07 0.06,0.08) | ( 0.09 0.07,0.11) | ( 0.04 0.03,0.05) |
|  | **20 to 24** | ( 624.06 503.58,769.35) | ( 237.30 191.24,285.70) | ( 357.96 285.40,464.81) | ( 151.15 123.63,182.50) | ( 0.39 0.32,0.48) | ( 0.15 0.12,0.19) | ( 0.20 0.16,0.26) | ( 0.09 0.07,0.11) |
|  | **25 to 29** | ( 1400.64 1156.66,1722.65) | ( 397.85 314.19,494.55) | ( 1256.76 1034.64,1576.29) | ( 294.85 233.92,370.43) | ( 1.02 0.84,1.25) | ( 0.30 0.24,0.37) | ( 0.70 0.57,0.87) | ( 0.17 0.14,0.22) |
|  | **30 to 35** | ( 2977.10 2489.77,3579.10) | ( 573.26 449.80,700.49) | ( 3814.15 3097.97,4943.64) | ( 576.21 450.81,720.63) | ( 2.55 2.13,3.06) | ( 0.52 0.41,0.63) | ( 1.98 1.61,2.57) | ( 0.32 0.25,0.40) |
|  | **35 to 39** | ( 5643.33 4713.92,6732.12) | ( 866.90 671.27,1093.31) | ( 6428.22 5106.41,8304.53) | ( 824.55 632.89,1059.38) | ( 5.16 4.31,6.16) | ( 0.84 0.65,1.06) | ( 3.64 2.89,4.71) | ( 0.49 0.38,0.63) |
|  | **40 to 44** | ( 7938.98 6461.76,9588.34) | ( 1072.45 818.47,1352.72) | ( 9520.34 7400.47,12136.26) | ( 1147.51 858.90,1493.36) | ( 9.13 7.43,11.03) | ( 1.33 1.01,1.68) | ( 6.13 4.77,7.82) | ( 0.77 0.58,1.01) |
|  | **45 to 49** | ( 8903.18 7245.88,10799.14) | ( 1315.43 1043.32,1660.63) | ( 15906.35 12167.98,20732.86) | ( 1975.42 1522.81,2559.14) | ( 12.66 10.31,15.36) | ( 2.03 1.61,2.56) | ( 10.50 8.03,13.69) | ( 1.35 1.04,1.75) |
|  | **50 to 54** | ( 10593.21 8651.92,12766.50) | ( 1647.05 1292.16,2109.66) | ( 21236.90 16083.98,28156.37) | ( 2958.05 2187.75,3922.93) | ( 17.09 13.96,20.59) | ( 2.87 2.25,3.68) | ( 14.81 11.22,19.64) | ( 2.10 1.55,2.79) |
|  | **55 to 59** | ( 10950.93 8916.01,13045.11) | ( 1942.94 1450.29,2573.91) | ( 20690.53 15744.41,27534.06) | ( 3417.66 2365.49,4747.04) | ( 20.38 16.59,24.28) | ( 3.85 2.88,5.10) | ( 16.85 12.82,22.42) | ( 2.76 1.91,3.84) |
|  | **60 to 64** | ( 9920.11 7893.87,12079.63) | ( 2076.65 1462.94,2721.58) | ( 17871.68 13010.96,24135.63) | ( 3471.90 2269.82,4833.16) | ( 22.88 18.20,27.86) | ( 5.00 3.52,6.55) | ( 19.24 14.01,25.99) | ( 3.66 2.39,5.10) |
|  | **65 to 69** | ( 7767.31 6149.81,9526.13) | ( 1976.74 1381.00,2658.76) | ( 17875.51 13139.24,23132.68) | ( 4190.09 2951.89,5614.13) | ( 25.20 19.96,30.91) | ( 6.19 4.32,8.32) | ( 21.93 16.12,28.39) | ( 4.90 3.45,6.56) |
|  | **70 to 74** | ( 5173.93 3974.79,6475.82) | ( 1541.29 1086.05,2105.76) | ( 12547.89 9204.06,16561.53) | ( 3321.54 2293.87,4535.45) | ( 25.76 19.79,32.25) | ( 6.79 4.79,9.28) | ( 21.74 15.95,28.69) | ( 5.32 3.67,7.26) |
|  | **75 to 79** | ( 2721.18 2178.08,3339.66) | ( 1023.99 743.36,1352.71) | ( 8140.43 6175.72,10432.88) | ( 2435.45 1681.71,3333.67) | ( 22.79 18.24,27.97) | ( 6.89 5.00,9.11) | ( 23.04 17.48,29.53) | ( 6.01 4.15,8.22) |
|  | **80 +** | ( 1495.69 1176.04,1870.29) | ( 723.24 511.43,979.40) | ( 9038.99 6908.14,11686.79) | ( 3347.32 2333.51,4604.67) | ( 17.57 13.81,21.97) | ( 5.79 4.10,7.85) | ( 27.84 21.28,36) | ( 6.97 4.86,9.58) |
| **Deaths (95% UI), per 100,000 population** | **<5** | ( 0 0,0) | ( 0 0,0) | ( 0 0,0) | ( 0 0,0) | ( 0 0,0) | ( 0 0,0) | ( 0 0,0) | ( 0 0,0) |
|  | **5 to 9** | ( 0 0,0) | ( 0 0,0) | ( 0 0,0) | ( 0 0,0) | ( 0 0,0) | ( 0 0,0) | ( 0 0,0) | ( 0 0,0) |
|  | **10 to 14** | ( 213.91 182.35,255.79) | ( 80.10 65.22,97.51) | ( 100.86 83.22,126.81) | ( 48.62 38.02,60.83) | ( 0.13 0.11,0.15) | ( 0.05 0.04,0.06) | ( 0.05 0.04,0.07) | ( 0.03 0.02,0.03) |
|  | **15 to 19** | ( 306.02 256.33,368.94) | ( 105.08 85.91,128.49) | ( 143.73 120.34,184.61) | ( 65.98 53.02,83.82) | ( 0.18 0.15,0.22) | ( 0.07 0.05,0.08) | ( 0.08 0.07,0.10) | ( 0.04 0.03,0.05) |
|  | **20 to 24** | ( 632.37 511.96,783.74) | ( 239.05 192.73,288.87) | ( 320.37 256.60,416.22) | ( 139.43 113.81,169.14) | ( 0.40 0.32,0.49) | ( 0.16 0.13,0.19) | ( 0.18 0.14,0.23) | ( 0.08 0.07,0.10) |
|  | **25 to 29** | ( 1296.22 1068.97,1596.48) | ( 365.32 288.70,455.27) | ( 1003.32 831.81,1247.51) | ( 240.57 190.85,298.85) | ( 0.94 0.78,1.16) | ( 0.27 0.22,0.34) | ( 0.56 0.46,0.69) | ( 0.14 0.11,0.18) |
|  | **30 to 35** | ( 2714.12 2270.59,3280.26) | ( 517.74 409.05,631.86) | ( 2961.45 2413.69,3832.05) | ( 450.32 356.66,557.57) | ( 2.32 1.94,2.80) | ( 0.47 0.37,0.57) | ( 1.54 1.25,1.99) | ( 0.25 0.20,0.31) |
|  | **35 to 39** | ( 4870.41 4070.92,5808.05) | ( 742.09 576.47,938.68) | ( 4737.05 3779.28,6128.14) | ( 609.23 468.64,787.10) | ( 4.46 3.72,5.31) | ( 0.72 0.56,0.91) | ( 2.68 2.14,3.47) | ( 0.36 0.28,0.47) |
|  | **40 to 44** | ( 7119.00 5771.31,8585.22) | ( 956.41 733.73,1201.72) | ( 7146.18 5526.12,9140.75) | ( 880.43 662.70,1133.29) | ( 8.19 6.64,9.87) | ( 1.19 0.91,1.49) | ( 4.60 3.56,5.89) | ( 0.59 0.45,0.76) |
|  | **45 to 49** | ( 8153.34 6618.71,9855.08) | ( 1205.90 955.33,1530.46) | ( 12080.55 9241.92,15771.36) | ( 1553.17 1202.07,2028.68) | ( 11.60 9.42,14.02) | ( 1.86 1.47,2.36) | ( 7.98 6.10,10.41) | ( 1.06 0.82,1.39) |
|  | **50 to 54** | ( 9827.92 8036.32,11885.57) | ( 1529.42 1197.26,1968.24) | ( 16460.68 12545.67,21959.52) | ( 2359.07 1747.91,3127.19) | ( 15.85 12.96,19.17) | ( 2.67 2.09,3.43) | ( 11.48 8.75,15.32) | ( 1.68 1.24,2.22) |
|  | **55 to 59** | ( 10489.12 8548.39,12485.08) | ( 1865.31 1392.52,2470.27) | ( 17274.25 13101.66,23158.03) | ( 2922.79 2027.06,4075.35) | ( 19.52 15.91,23.24) | ( 3.70 2.76,4.90) | ( 14.06 10.67,18.86) | ( 2.36 1.64,3.29) |
|  | **60 to 64** | ( 9764.67 7777.43,11885.85) | ( 2044.49 1437.23,2671.41) | ( 15422.01 11056.38,20850.57) | ( 3068.27 1991.48,4305.99) | ( 22.52 17.93,27.41) | ( 4.92 3.46,6.43) | ( 16.61 11.90,22.45) | ( 3.23 2.10,4.54) |
|  | **65 to 69** | ( 7987.86 6300.16,9780.06) | ( 2022.45 1407.23,2709.55) | ( 16271.65 11953.93,21060.17) | ( 3918.67 2769.93,5263.53) | ( 25.92 20.44,31.74) | ( 6.33 4.41,8.48) | ( 19.97 14.67,25.84) | ( 4.58 3.24,6.15) |
|  | **70 to 74** | ( 5593.64 4294.11,7006.42) | ( 1659.78 1163.68,2277.56) | ( 11909.17 8782.86,15598.66) | ( 3241.75 2219.45,4376.06) | ( 27.85 21.38,34.89) | ( 7.32 5.13,10.04) | ( 20.63 15.22,27.02) | ( 5.19 3.55,7.01) |
|  | **75 to 79** | ( 3124.22 2497.94,3847.88) | ( 1172.01 852.68,1549.90) | ( 8248.94 6271.89,10630.80) | ( 2519.86 1734.54,3441.62) | ( 26.16 20.92,32.22) | ( 7.89 5.74,10.44) | ( 23.35 17.75,30.09) | ( 6.21 4.28,8.49) |
|  | **80 +** | ( 1876.38 1483.33,2352.27) | ( 904.09 637.95,1231.67) | ( 9951.03 7622.31,12772.47) | ( 3693.37 2596.86,5041.98) | ( 22.04 17.42,27.63) | ( 7.24 5.11,9.87) | ( 30.65 23.48,39.34) | ( 7.69 5.40,10.49) |
| **Prevalence (95% UI), per 100,000 population** | **<5** | ( 0 0,0) | ( 0 0,0) | ( 0 0,0) | ( 0 0,0) | ( 0 0,0) | ( 0 0,0) | ( 0 0,0) | ( 0 0,0) |
|  | **5 to 9** | ( 0 0,0) | ( 0 0,0) | ( 0 0,0) | ( 0 0,0) | ( 0 0,0) | ( 0 0,0) | ( 0 0,0) | ( 0 0,0) |
|  | **10 to 14** | ( 377.65 321.10,454.71) | ( 124.28 98.80,153.53) | ( 192.01 155.87,248.98) | ( 80.42 62.38,102.00) | ( 0.22 0.19,0.27) | ( 0.08 0.06,0.10) | ( 0.10 0.08,0.13) | ( 0.05 0.04,0.06) |
|  | **15 to 19** | ( 519.83 435.81,624.93) | ( 189.98 155.47,231.45) | ( 283.12 236.04,361.62) | ( 136.41 110.53,170.40) | ( 0.31 0.26,0.37) | ( 0.12 0.10,0.15) | ( 0.15 0.13,0.20) | ( 0.08 0.07,0.10) |
|  | **20 to 24** | ( 970.16 783.06,1195.72) | ( 399.43 322.24,479.88) | ( 583.15 463.98,757.11) | ( 273.36 224.47,329.14) | ( 0.61 0.49,0.75) | ( 0.26 0.21,0.31) | ( 0.33 0.26,0.42) | ( 0.16 0.13,0.20) |
|  | **25 to 29** | ( 3365.59 2782.79,4137.60) | ( 884.60 699.24,1098.28) | ( 3163.09 2601.72,3959.64) | ( 709.44 558.91,888.59) | ( 2.45 2.02,3.01) | ( 0.66 0.53,0.83) | ( 1.75 1.44,2.20) | ( 0.42 0.33,0.52) |
|  | **30 to 35** | ( 6264.32 5241.50,7531.85) | ( 1218.02 957.44,1487.08) | ( 8412.90 6824.55,10926.01) | ( 1344.17 1041.37,1690.26) | ( 5.36 4.48,6.44) | ( 1.10 0.87,1.35) | ( 4.37 3.55,5.68) | ( 0.74 0.57,0.93) |
|  | **35 to 39** | ( 9531.08 7965.62,11361.31) | ( 1467.69 1137.24,1852.73) | ( 11512.46 9127.15,14823.86) | ( 1565.35 1208.79,2015.04) | ( 8.72 7.29,10.40) | ( 1.42 1.10,1.80) | ( 6.52 5.17,8.40) | ( 0.93 0.72,1.20) |
|  | **40 to 44** | ( 10315.90 8431.19,12447.04) | ( 1435.17 1102.13,1803.58) | ( 14233.36 11160.66,18260.27) | ( 1959.63 1466.18,2580.53) | ( 11.86 9.70,14.31) | ( 1.78 1.37,2.24) | ( 9.17 7.19,11.76) | ( 1.32 0.99,1.74) |
|  | **45 to 49** | ( 11705.15 9522.11,14159.41) | ( 1698.33 1349.30,2136.71) | ( 24372.66 18669.56,31474.49) | ( 3129.03 2390.72,4086.41) | ( 16.65 13.55,20.14) | ( 2.62 2.08,3.29) | ( 16.09 12.33,20.78) | ( 2.14 1.63,2.79) |
|  | **50 to 54** | ( 13685.10 11248.72,16452.34) | ( 2114.66 1654.51,2705.02) | ( 32076.60 24389.32,42370.74) | ( 4518.48 3351.58,6058.86) | ( 22.07 18.14,26.54) | ( 3.69 2.89,4.72) | ( 22.38 17.01,29.56) | ( 3.21 2.38,4.31) |
|  | **55 to 59** | ( 13731.61 11208.14,16387.50) | ( 2386.28 1781.02,3153.79) | ( 29997.51 22825.39,40059.64) | ( 4825.64 3319.90,6713.59) | ( 25.56 20.86,30.50) | ( 4.73 3.53,6.25) | ( 24.42 18.58,32.62) | ( 3.90 2.68,5.43) |
|  | **60 to 64** | ( 11407.98 9095.29,13825.46) | ( 2344.32 1665.81,3073.51) | ( 24726.69 17966.50,33248.03) | ( 4571.68 2963.49,6405.14) | ( 26.31 20.97,31.88) | ( 5.64 4.01,7.39) | ( 26.62 19.35,35.80) | ( 4.82 3.12,6.75) |
|  | **65 to 69** | ( 8335.11 6615.87,10204.16) | ( 2079.10 1467.05,2795.75) | ( 23194.16 17116.01,29855.13) | ( 5025.04 3572.15,6750.88) | ( 27.05 21.47,33.11) | ( 6.51 4.59,8.75) | ( 28.46 21.00,36.63) | ( 5.87 4.17,7.89) |
|  | **70 to 74** | ( 4750.25 3662.48,5944.10) | ( 1505.46 1068.32,2054.50) | ( 14488.22 10536.63,19009.82) | ( 3857.12 2638.28,5231.79) | ( 23.65 18.24,29.60) | ( 6.64 4.71,9.06) | ( 25.10 18.25,32.93) | ( 6.18 4.22,8.38) |
|  | **75 to 79** | ( 2301.63 1838.55,2828.50) | ( 890.54 648.69,1181.67) | ( 9108.30 6955.24,11809.05) | ( 2655.05 1817.38,3701.53) | ( 19.27 15.40,23.69) | ( 6.00 4.37,7.96) | ( 25.78 19.69,33.42) | ( 6.55 4.48,9.13) |
|  | **80 +** | ( 1079.87 843.21,1349.18) | ( 545.32 384.96,731.47) | ( 9263.32 7090.86,11788.79) | ( 3375.79 2256.17,4636.46) | ( 12.69 9.91,15.85) | ( 4.37 3.08,5.86) | ( 28.53 21.84,36.31) | ( 7.02 4.69,9.65) |
| **DALYs (95% UI), per 100,000 population** | **<5** | ( 0 0,0) | ( 0 0,0) | ( 0 0,0) | ( 0 0,0) | ( 0 0,0) | ( 0 0,0) | ( 0 0,0) | ( 0 0,0) |
|  | **5 to 9** | ( 0 0,0) | ( 0 0,0) | ( 0 0,0) | ( 0 0,0) | ( 0 0,0) | ( 0 0,0) | ( 0 0,0) | ( 0 0,0) |
|  | **10 to 14** | ( 16630.89 14175.58,19881.02) | ( 6225.58 5067.74,7577.62) | ( 7835.18 6463.24,9853.59) | ( 3777.76 2953.92,4726.53) | ( 9.86 8.40,11.78) | ( 3.93 3.20,4.79) | ( 4.04 3.33,5.08) | ( 2.13 1.66,2.66) |
|  | **15 to 19** | ( 22267.84 18649.72,26839.72) | ( 7647.10 6254.93,9356.95) | ( 10455.25 8757.28,13426.27) | ( 4804.60 3861.67,6097.64) | ( 13.31 11.15,16.05) | ( 4.79 3.92,5.87) | ( 5.68 4.76,7.30) | ( 2.84 2.28,3.60) |
|  | **20 to 24** | ( 42966.93 34811.65,53247.21) | ( 16235.24 13098.69,19634.97) | ( 21742.94 17408.85,28261.56) | ( 9469.57 7729.90,11489.00) | ( 27.03 21.90,33.49) | ( 10.57 8.53,12.79) | ( 12.20 9.77,15.85) | ( 5.64 4.60,6.84) |
|  | **25 to 29** | ( 81828.39 67470.42,100758.53) | ( 23052.68 18215.47,28731.39) | ( 63173.04 52376.25,78488.75) | ( 15153.66 12021.57,18833.14) | ( 59.48 49.04,73.24) | ( 17.32 13.69,21.59) | ( 35.05 29.06,43.55) | ( 8.87 7.04,11.03) |
|  | **30 to 35** | ( 157045.07 131417.10,189826.45) | ( 29985.15 23680.30,36575.02) | ( 171673.01 139783.16,222170.66) | ( 26119.09 20705.71,32338.05) | ( 134.27 112.36,162.30) | ( 27.13 21.43,33.10) | ( 89.23 72.65,115.47) | ( 14.33 11.36,17.74) |
|  | **35 to 39** | ( 258437.28 216222.36,308336.47) | ( 39387.93 30602.99,49787.43) | ( 251814.70 200905.28,325666.62) | ( 32373.11 24889.58,41827.76) | ( 236.47 197.84,282.13) | ( 38.19 29.67,48.27) | ( 142.69 113.84,184.54) | ( 19.30 14.84,24.94) |
|  | **40 to 44** | ( 342661.55 277946.49,413470.17) | ( 46069.44 35363.75,57877.58) | ( 344001.90 265841.69,439803.05) | ( 42380.43 31879.29,54630.14) | ( 394.06 319.64,475.49) | ( 57.12 43.85,71.76) | ( 221.58 171.24,283.29) | ( 28.58 21.50,36.84) |
|  | **45 to 49** | ( 352141.13 285893.77,425922.27) | ( 52059.21 41238.23,66062.88) | ( 521597.09 398825.46,680112.38) | ( 67050.08 51964.33,87534.24) | ( 500.93 406.69,605.88) | ( 80.21 63.54,101.79) | ( 344.36 263.31,449.01) | ( 45.81 35.50,59.80) |
|  | **50 to 54** | ( 377315.26 308237.16,456312.40) | ( 58700.42 45943.06,75485.77) | ( 633151.16 482454.40,844945.29) | ( 90652.46 67143.65,120075.93) | ( 608.59 497.17,736.01) | ( 102.41 80.16,131.70) | ( 441.66 336.54,589.40) | ( 64.41 47.71,85.32) |
|  | **55 to 59** | ( 354133.04 288811.90,421804.29) | ( 62951.47 47013.56,83507.54) | ( 584343.29 443224.77,784522.02) | ( 98813.57 68448.34,137943.22) | ( 659.12 537.54,785.07) | ( 124.80 93.21,165.56) | ( 475.77 360.87,638.76) | ( 79.87 55.33,111.50) |
|  | **60 to 64** | ( 284337.57 226521.37,346537.05) | ( 59477.24 41835.09,77656.49) | ( 448906.14 321973.78,606900.29) | ( 89231.21 57928.76,125371.61) | ( 655.68 522.36,799.12) | ( 143.09 100.65,186.83) | ( 483.35 346.68,653.47) | ( 94.08 61.08,132.18) |
|  | **65 to 69** | ( 195864.96 154539.62,239697.47) | ( 49525.11 34475.73,66339.51) | ( 399245.71 293481.52,518060.86) | ( 95970.49 67893.79,129014.87) | ( 635.56 501.46,777.79) | ( 155.06 107.94,207.70) | ( 489.91 360.13,635.70) | ( 112.14 79.33,150.75) |
|  | **70 to 74** | ( 112978.76 86791.39,141589.30) | ( 33460.06 23485.42,46000.74) | ( 240755.10 177393.36,315481.40) | ( 65524.78 44813.17,88664.57) | ( 562.57 432.17,705.03) | ( 147.49 103.52,202.77) | ( 417.10 307.33,546.56) | ( 104.92 71.75,141.97) |
|  | **75 to 79** | ( 50694.04 40551.83,62567.54) | ( 18954.76 13777.52,25016.71) | ( 133421.57 101272.67,172067.66) | ( 40627.51 27971.01,55354.70) | ( 424.52 339.59,523.95) | ( 127.62 92.76,168.43) | ( 377.62 286.63,486.99) | ( 100.18 68.97,136.50) |
|  | **80 +** | ( 22270.09 17687.24,27944.93) | ( 10536.50 7456.67,14304.82) | ( 114055.72 87313.30,146238.95) | ( 41611.99 29055.99,56925.48) | ( 261.61 207.77,328.27) | ( 84.41 59.74,114.60) | ( 351.31 268.94,450.44) | ( 86.59 60.46,118.45) |

**Supplementary Table 3: Number and rate of incidence, deaths, prevalence and DALYs of HBV associated liver cancer in different age groups and regions in Asia in 1990 and 2021.**

| **Region** | **Age** | **Incidence** | | | | **Prevalence** | | | | **Deaths** | | | | **DALYs** | | | |
| --- | --- | --- | --- | --- | --- | --- | --- | --- | --- | --- | --- | --- | --- | --- | --- | --- | --- |
|  |  | **1990Number** | **2021Number** | **1990Rate** | **2021Rate** | **1990Number** | **2021Number** | **1990Rate** | **2021Rate** | **1990Number** | **2021Number** | **1990Rate** | **2021Rate** | **1990Number** | **2021Number** | **1990Rate** | **2021Rate** |
| **Central Asia** | **0 to 14** | ( 4.86 3.30,6.91) | ( 2.91 1.88,4.23) | ( 0.02 0.01,0.03) | ( 0.01 0.01,0.02) | ( 9.03 6.13,12.82) | ( 5.44 3.52,7.89) | ( 0.04 0.02,0.05) | ( 0.02 0.01,0.03) | ( 5.81 4.22,7.83) | ( 3.69 2.58,5.18) | ( 0.02 0.02,0.03) | ( 0.01 0.01,0.02) | ( 451.33 327.66,608.70) | ( 286.56 200.73,402.98) | ( 1.81 1.31,2.44) | ( 1.04 0.73,1.46) |
|  | **15 to 49** | ( 257.12 193.01,320.85) | ( 332.00 232.70,445.15) | ( 0.77 0.58,0.96) | ( 0.68 0.48,0.91) | ( 404.40 307.37,499.06) | ( 505.17 355.22,675.00) | ( 1.21 0.92,1.50) | ( 1.04 0.73,1.38) | ( 239.42 179.52,298.55) | ( 306.82 215.14,411.87) | ( 0.72 0.54,0.90) | ( 0.63 0.44,0.84) | ( 12457.55 9436.75,15461.02) | ( 15365.43 10831.24,20552.50) | ( 37.36 28.30,46.36) | ( 31.51 22.21,42.15) |
|  | **50 to 69** | ( 652.96 422.56,940.31) | ( 876.67 536.18,1349.49) | ( 7.49 4.85,10.78) | ( 5.49 3.36,8.45) | ( 745.42 486.03,1073.75) | ( 1007.32 620.55,1548.75) | ( 8.55 5.57,12.31) | ( 6.31 3.88,9.69) | ( 651.59 421.32,942.34) | ( 874.62 530.44,1357.50) | ( 7.47 4.83,10.81) | ( 5.47 3.32,8.50) | ( 20796.89 13580.35,30259.13) | ( 27490.14 16900.77,42554.68) | ( 238.48 155.72,346.98) | ( 172.08 105.79,266.38) |
|  | **70+** | ( 137.33 80.71,218.32) | ( 187.35 106.13,311.84) | ( 6.09 3.58,9.69) | ( 5.51 3.12,9.17) | ( 112.12 65.80,179.54) | ( 153.19 86.93,255.77) | ( 4.98 2.92,7.97) | ( 4.51 2.56,7.52) | ( 161.03 94.73,255.58) | ( 219.94 125.66,364.35) | ( 7.15 4.20,11.34) | ( 6.47 3.70,10.72) | ( 2698.51 1582.28,4345.41) | ( 3619.82 2067.24,6081.22) | ( 119.76 70.22,192.85) | ( 106.49 60.82,178.90) |
| **East Asia** | **0 to 14** | ( 174.84 142.16,215.46) | ( 67.00 51.90,89.12) | ( 0.05 0.04,0.07) | ( 0.03 0.02,0.03) | ( 325.42 264.71,401.14) | ( 134.73 104.62,178.90) | ( 0.10 0.08,0.12) | ( 0.05 0.04,0.07) | ( 182.30 148.49,224.32) | ( 58.69 45.62,77.64) | ( 0.06 0.05,0.07) | ( 0.02 0.02,0.03) | ( 14166.49 11540.71,17437.30) | ( 4559.78 3545.05,6027.18) | ( 4.30 3.50,5.29) | ( 1.71 1.33,2.25) |
|  | **15 to 49** | ( 25112.45 20777.85,30080.11) | ( 31737.22 24580.65,41527.04) | ( 3.65 3.02,4.37) | ( 4.61 3.57,6.03) | ( 38483.88 31963.97,46071.82) | ( 53334.91 41770.03,69458.16) | ( 5.59 4.64,6.69) | ( 7.75 6.07,10.09) | ( 22757.45 18823.46,27237.72) | ( 23263.14 18044.48,30485.29) | ( 3.30 2.73,3.95) | ( 3.38 2.62,4.43) | ( 1147442.58 952550.35,1371699.54) | ( 1132006.93 880933.95,1482229.15) | ( 166.57 138.28,199.13) | ( 164.42 127.95,215.28) |
|  | **50 to 69** | ( 31465.04 25574.06,38091.54) | ( 64486.94 48483.03,85038.30) | ( 19.69 16.00,23.83) | ( 16.39 12.33,21.62) | ( 36840.63 29962.97,44642.64) | ( 90317.33 67849.44,119632.98) | ( 23.05 18.75,27.93) | ( 22.96 17.25,30.41) | ( 30866.01 25080.19,37228.86) | ( 53350.12 40086.43,70177.92) | ( 19.31 15.69,23.29) | ( 13.56 10.19,17.84) | ( 977658.21 795768.44,1179499.86) | ( 1679082.63 1264449.64,2225351.02) | ( 611.65 497.86,737.93) | ( 426.88 321.46,565.76) |
|  | **70+** | ( 8335.82 6818.49,10214.68) | ( 25888.38 19763.12,33397.15) | ( 21.42 17.52,26.25) | ( 20.95 15.99,27.02) | ( 7046.94 5754.56,8653.79) | ( 26417.22 19959.58,34150.37) | ( 18.11 14.79,22.24) | ( 21.38 16.15,27.63) | ( 9519.35 7778.93,11647.49) | ( 26685.92 20516.29,34382.11) | ( 24.46 19.99,29.93) | ( 21.59 16.60,27.82) | ( 166769.13 135656.08,205627.85) | ( 431402.65 328873.08,559563.22) | ( 428.56 348.61,528.42) | ( 349.09 266.12,452.80) |
| **High-income Asia Pacific** | **0 to 14** | ( 13.54 8.65,20.59) | ( 2.77 2.13,3.67) | ( 0.04 0.02,0.06) | ( 0.01 0.01,0.02) | ( 27.83 18.34,41.54) | ( 7.85 6.05,10.41) | ( 0.08 0.05,0.12) | ( 0.04 0.03,0.05) | ( 12.94 8.12,19.64) | ( 1.75 1.38,2.27) | ( 0.04 0.02,0.06) | ( 0.01 0.01,0.01) | ( 1005.95 631.28,1526.93) | ( 135.80 107.74,176.74) | ( 2.86 1.79,4.34) | ( 0,61 0.48,0.79) |
|  | **15 to 49** | ( 2542.02 1910.31,3404.40) | ( 1529.73 1172.26,2018.61) | ( 2.74 2.06,3.67) | ( 1.96 1.50,2.58) | ( 4229.17 3285.75,5491.72) | ( 3940.30 3040.25,5163.64) | ( 4.56 3.54,5.92) | ( 5.04 3.89,6.60) | ( 2094.40 1544.91,2817.40) | ( 839.18 644.54,1099.19) | ( 2.26 1.66,3.04) | ( 1.07 0.82,1.41) | ( 100973.63 75189.02,134419.16) | ( 39344.86 30510.57,51166.74) | ( 108.78 81.00,144.81) | ( 50.30 39.01,65.41) |
|  | **50 to 69** | ( 6705.85 5108.39,8183.36) | ( 6986.00 5311.58,8963.34) | ( 19.66 14.98,24.00) | ( 14.01 10.65,17.98) | ( 9278.44 7286.28,11333.52) | ( 14271.90 10866.31,17945.25) | ( 27.21 21.37,33.24) | ( 28.62 21.79,35.99) | ( 5971.17 4509.65,7369.63) | ( 4675.41 3544.45,6005.20) | ( 17.51 13.22,21.61) | ( 9.38 7.11,12.04) | ( 190475.87 145404.92,234601.54) | ( 143996.26 109844.72,184406.41) | ( 558.57 426.40,687.97) | ( 288.79 220.30,369.83) |
|  | **70+** | ( 2037.46 1469.97,2630.59) | ( 6299.83 4621.56,8128.71) | ( 18.10 13.06,23.37) | ( 18.03 13.23,23.27) | ( 2120.32 1553.61,2698.27) | ( 10613.96 7729.97,13854.37) | ( 18.84 13.80,23.97) | ( 30.38 22.12,39.65) | ( 2126.64 1520.45,2752.96) | ( 5222.48 3849.54,6796.25) | ( 18.89 13.51,24.46) | ( 14.95 11.02,19.45) | ( 35980.44 25576.38,47040.73) | ( 77280.00 56685.61,100462.73) | ( 319.66 227.23,417.93) | ( 221.18 162.24,287.54) |
| **North Africa and Middle East** | **0 to 14** | ( 18.31 12.12,26.09) | ( 15.75 10.04,23.58) | ( 0.01 0.01,0.02) | ( 0.01 0.01,0.01) | ( 34.02 22.54,48.46) | ( 30.17 19.36,45.07) | ( 0.02 0.02,0.03) | ( 0.02 0.01,0.02) | ( 22.21 15.28,31.20) | ( 19.79 13.50,28.36) | ( 0.02 0.01,0.02) | ( 0.01 0.01,0.02) | ( 1728.01 1189.31,2427.81) | ( 1538.02 1048.89,2204.57) | ( 1.23 0,85,1,73) | ( 0.84 0.57,1.20) |
|  | **15 to 49** | ( 609.18 457.97,826.24) | ( 1454.34 1144.21,1835.24) | ( 0.38 0.29,0.52) | ( 0.43 0.34,0.55) | ( 932.61 706.49,1259.93) | ( 2315.80 1829.90,2885.44) | ( 0.58 0.44,0.79) | ( 0.69 0.55,0.86) | ( 563.35 422.45,764.10) | ( 1276.58 1001.18,1612.24) | ( 0.35 0.26,0.48) | ( 0.38 0.30,0.48) | ( 28813.57 21705.52,38994.54) | ( 64008.75 50397.42,80084.33) | ( 17.98 13.54,24.33) | ( 19.15 15.07,23.95) |
|  | **50 to 69** | ( 1367.82 942.28,1988.11) | ( 3598.94 2400.77,5038.14) | ( 4.38 3.02,6.37) | ( 4.23 2.82,5.93) | ( 1562.76 1081.04,2272.46) | ( 4320.82 2928.69,6023.74) | ( 5.01 3.46,7.28) | ( 5.08 3.45,7.09) | ( 1368.00 942.54,1981.46) | ( 3499.86 2321.62,4911.69) | ( 4.38 3.02,6.35) | ( 4.12 2.73,5.78) | ( 42746.24 29499.15,62139.52) | ( 109434.52 74576.97,152508.62) | ( 136.95 94.51,199.09) | ( 128.73 87.72,179.39) |
|  | **70+** | ( 413.29 259.30,662.20) | ( 1234.71 824.56,1831.34) | ( 5.72 3.59,9.17) | ( 6.07 4.06,9.01) | ( 342.58 213.37,547.81) | ( 1066.97 712.43,1580.86) | ( 4.74 2.95,7.59) | ( 5.25 3.50,7.77) | ( 481.87 302.62,769.01) | ( 1422.95 960.20,2101.82) | ( 6.67 4.19,10.65) | ( 7.00 4.72,10.34) | ( 8259.88 5128.75,13195.37) | ( 23564.04 15722.51,35371.65) | ( 114.38 71.02,182.72) | ( 115.88 77.32,173.95) |
| **South Asia** | **0 to 14** | ( 31.14 23.08,40.46) | ( 38.41 29.08,49.83) | ( 0.01 0.01,0.01) | ( 0.01 0.01,0.01) | ( 57.41 42.57,74.64) | ( 71.68 54.25,92.97) | ( 0.01 0.01,0.02) | ( 0.01 0.01,0.02) | ( 40.89 31.59,51.56) | ( 52.71 40.74,67.05) | ( 0.01 0.01,0.01) | ( 0.01 0.01,0.01) | ( 3181.40 2457.46,4011.25) | ( 4094.05 3164.52,5208.48) | ( 0.73 0.57,0.93) | ( 0.81 0.62,1.03) |
|  | **15 to 49** | ( 1460.27 1237.74,1750.66) | ( 3586.38 2908.66,4400.29) | ( 0.28 0.23,0.33) | ( 0.36 0.29,0.44) | ( 2230.56 1912.46,2663.34) | ( 5530.81 4517.96,6785.22) | ( 0.42 0.36,0.50) | ( 0.55 0.45,0.67) | ( 1359.48 1155.07,1628.25) | ( 3272.26 2653.85,4006.61) | ( 0.26 0.22,0.31) | ( 0.33 0.26,0.40) | ( 69637.18 59645.99,82793.58) | ( 165607.12 134871.08,202657.39) | ( 13.16 11.27,15.65) | ( 16.45 13.40,20.13) |
|  | **50 to 69** | ( 2659.48 2116.09,3309.58) | ( 7388.21 5755.79,9250.82) | ( 2.47 1.97,3.08) | ( 2.85 2.22,3.56) | ( 3004.12 2386.35,3753.40) | ( 8511.09 6627.83,10695.05) | ( 2.79 2.22,3.49) | ( 3.28 2.55,4.12) | ( 2670.07 2124.42,3325.27) | ( 7363.60 5750.70,9224.27) | ( 2.48 1.98,3.09) | ( 2.84 2.22,3.55) | ( 83679.62 66571.32,104959.88) | ( 227779.58 177898.19,286483.31) | ( 77.82 61.91,97.61) | ( 87.73 68.52,110.35) |
|  | **70+** | ( 735.41 572.50,928.59) | ( 2991.24 2344.00,3699.74) | ( 3.13 2.44,3.95) | ( 4.09 3.20,5.05) | ( 600.06 464.45,759.91) | ( 2498.29 1948.42,3095.89) | ( 2.55 1.98,3.24) | ( 3.41 2.66,4.23) | ( 853.90 664.30,1076.76) | ( 3483.63 2751.75,4306.89) | ( 3.64 2.83,4.58) | ( 4.76 3.76,5.88) | ( 14941.91 11512.81,18897.01) | ( 59048.19 46024.65,73252.46) | ( 63.62 49.02,80.45) | ( 80.65 62.86,100.05) |
| **Southeast Asia** | **0 to 14** | ( 34.65 25.33,46.09) | ( 18.27 13.30,25.69) | ( 0.02 0.01,0.03) | ( 0.01 0.01,0.01) | ( 64.27 46.97,85.63) | ( 34.92 25.35,49.29) | ( 0.04 0.03,0.05) | ( 0.02 0.01,0.03) | ( 40.69 31.01,52.22) | ( 21.83 16.65,29.71) | ( 0.02 0.02,0.03) | ( 0.01 0.01,0.02) | ( 3164.53 2412.03,4061.51) | ( 1696.58 1293.39,2308.05) | ( 1.85 1.41,2.38) | ( 0.98 0.75,1.34) |
|  | **15 to 49** | ( 2730.80 2238.95,3279.19) | ( 4508.95 3408.74,6124.08) | ( 1.15 0.95,1.39) | ( 1.22 0.92,1.65) | ( 4197.99 3470.22,4996.09) | ( 7040.84 5376.08,9512.52) | ( 1.77 1.47,2.11) | ( 1.90 1.45,2.57) | ( 2525.59 2072.69,3034.22) | ( 3973.70 2992.83,5436.64) | ( 1.07 0.88,1.28) | ( 1.07 0.81,1.47) | ( 128637.31 106031.29,153602.57) | ( 195586.42 149603.54,265757.45) | ( 54.37 44.82,64.92) | ( 52.75 40.35,71.67) |
|  | **50 to 69** | ( 4866.25 3551.09,6348.99) | ( 10375.60 7056.20,14888.28) | ( 10.30 7.52,13.44) | ( 8.32 5.66,11.93) | ( 5610.04 4099.1,7311.67) | ( 12772.28 8706.94,18275.84) | ( 11.87 8.68,15.48) | ( 10.24 6.98,14.65) | ( 4850.13 3539.52,6361.34) | ( 9933.26 6768.06,14125.66) | ( 10.27 7.49,13.46) | ( 7.96 5.42,11.32) | ( 153506.48 113063.99,200280.49) | ( 314757.01 215214.41,448097.42) | ( 324.91 239.31,423.91) | ( 252.27 172.49,359.14) |
|  | **70+** | ( 1250.85 867.38,1774.90) | ( 2797.96 1825.82,4122.07) | ( 11.46 7.95,16.26) | ( 9.30 6.07,13.70) | ( 1038.89 717.86,1470.67) | ( 2446.54 1586.77,3590.59) | ( 9.52 6.58,13.47) | ( 8.13 5.28,11.94) | ( 1458.98 1012.17,2050.54) | ( 3202.71 2091.52,4702.31) | ( 13.36 9.27,18.78) | ( 10.65 6.95,15.63) | ( 24846.29 17111.55,35003.45) | ( 52521.15 34204.18,76356.60) | ( 227.59 156.74,320.64) | ( 174.62 113.72,253.86) |

**Supplementary Table 4: Incidence, deaths, prevalence and DALYs rate of HBV associated liver cancer in Asian countries in 1990 and 2021.**

| **Country** | **Incidence (95% UI), per 100,000 population** | | **Prevalence (95% UI), per 100,000 population** | | **Mortality (95% UI), per 100,000 population** | | **DALYs (95% UI), per 100,000 population** | |
| --- | --- | --- | --- | --- | --- | --- | --- | --- |
|  | **1990** | **2021** | **1990** | **2021** | **1990** | **2021** | **1990** | **2021** |
| **Afghanistan** | ( 2.14 1.34,3.29) | ( 2.02 1.25,3.03) | ( 2.3 1.47,3.42) | ( 2.3 1.47,3.42) | ( 2.21 1.37,3.47) | ( 2.08 1.28,3.12) | ( 70.05 45.08,105.11) | ( 65.1 41.77,96.3) |
| **Armenia** | ( 1.85 1.24,2.58) | ( 1.32 0.87,1.87) | ( 1.52 1.02,2.12) | ( 1.52 1.02,2.12) | ( 1.93 1.28,2.7) | ( 1.35 0.89,1.93) | ( 56.28 38.89,77.69) | ( 40 27.11,55.93) |
| **Azerbaijan** | ( 1.62 0.87,2.7) | ( 1.71 0.85,3.17) | ( 1.93 0.98,3.53) | ( 1.93 0.98,3.53) | ( 1.66 0.89,2.76) | ( 1.76 0.85,3.29) | ( 52.75 28.89,84.98) | ( 52.84 26.7,96.14) |
| **Bahrain** | ( 2.6 1.71,3.7) | ( 1.35 0.88,2.02) | ( 1.53 0.99,2.27) | ( 1.53 0.99,2.27) | ( 2.75 1.82,3.93) | ( 1.39 0.91,2.08) | ( 72.07 47.94,102.67) | ( 35.29 22.86,52.7) |
| **Bangladesh** | ( 0.73 0.52,1.04) | ( 0.63 0.4,0.96) | ( 0.73 0.47,1.13) | ( 0.73 0.47,1.13) | ( 0.76 0.53,1.07) | ( 0.65 0.41,0.99) | ( 23.71 16.97,33.24) | ( 19.77 12.79,30.62) |
| **Bhutan** | ( 1.03 0.54,1.63) | ( 1.05 0.62,1.68) | ( 1.21 0.72,1.95) | ( 1.21 0.72,1.95) | ( 1.06 0.55,1.69) | ( 1.08 0.63,1.74) | ( 33.45 17.55,52.16) | ( 32.43 19.45,52.56) |
| **Brunei Darussalam** | ( 7.05 4.84,9.96) | ( 4.32 3.04,5.99) | ( 5.23 3.69,7.13) | ( 5.23 3.69,7.13) | ( 7.15 4.89,10.13) | ( 4.20 2.95,5.89) | ( 202.51 140.79,288.17) | ( 118.25 84,161.05) |
| **Cambodia** | ( 3.6 1.8,6.88) | ( 2.22 1.08,4.43) | ( 2.58 1.25,5.05) | ( 2.58 1.25,5.05) | ( 3.71 1.85,7.01) | ( 2.28 1.10,4.47) | ( 115.64 60,224.73) | ( 69.37 33.53,136.58) |
| **China** | ( 6.58 5.45,7.84) | ( 5.73 4.48,7.38) | ( 8.08 6.33,10.47) | ( 8.08 6.33,10.47) | ( 6.53 5.42,7.76) | ( 4.83 3.76,6.19) | ( 220.05 181.34,260.91) | ( 155.81 121.32,201.99) |
| **Cyprus** | ( 0.49 0.32,0.74) | ( 0.44 0.28,0.69) | ( 0.63 0.39,0.96) | ( 0.63 0.39,0.96) | ( 0.51 0.33,0.77) | ( 0.40 0.25,0.63) | ( 13.7 8.81,19.96) | ( 10.38 6.55,15.89) |
| **Democratic People's Republic of Korea** | ( 6.23 3.35,9.46) | ( 4.03 2.52,5.98) | ( 5.12 3.1,7.58) | ( 5.12 3.1,7.58) | ( 6.23 3.37,9.48) | ( 3.90 2.45,5.77) | ( 214.4 116.19,331.65) | ( 134.46 80.81,199.66) |
| **Georgia** | ( 1.22 0.89,1.68) | ( 0.93 0.63,1.3) | ( 1.08 0.74,1.51) | ( 1.08 0.74,1.51) | ( 1.23 0.9,1.7) | ( 0.94 0.63,1.33) | ( 39.72 29.57,53.46) | ( 29.6 20.3,41.17) |
| **India** | ( 0.78 0.64,0.93) | ( 0.95 0.8,1.14) | ( 1.08 0.92,1.3) | ( 1.08 0.92,1.3) | ( 0.81 0.66,0.96) | ( 0.98 0.82,1.17) | ( 24.97 20.77,29.46) | ( 29.3 24.78,35.13) |
| **Indonesia** | ( 0.87 0.58,1.32) | ( 1.03 0.61,1.65) | ( 1.23 0.75,1.98) | ( 1.23 0.75,1.98) | ( 0.88 0.59,1.34) | ( 1.04 0.61,1.66) | ( 29.56 20.06,44.8) | ( 33.33 20.32,53.68) |
| **Iran (Islamic Republic of)** | ( 0.99 0.8,1.28) | ( 1.22 1.06,1.41) | ( 1.42 1.23,1.62) | ( 1.42 1.23,1.62) | ( 1.04 0.84,1.35) | ( 1.26 1.08,1.45) | ( 29.28 23.79,37.36) | ( 34.07 29.99,38.7) |
| **Iraq** | ( 1.5 1,2.13) | ( 1.51 0.96,2.17) | ( 1.71 1.09,2.43) | ( 1.71 1.09,2.43) | ( 1.54 1.04,2.18) | ( 1.54 0.97,2.24) | ( 46.98 32.43,67.27) | ( 43.64 27.7,62.3) |
| **Israel** | ( 0.36 0.25,0.51) | ( 0.37 0.25,0.53) | ( 0.52 0.36,0.72) | ( 0.52 0.36,0.72) | ( 0.37 0.25,0.52) | ( 0.34 0.23,0.50) | ( 9.97 6.86,13.67) | ( 9.57 6.43,13.1) |
| **Japan** | ( 2.16 1.84,2.49) | ( 1.27 1.04,1.52) | ( 2.73 2.27,3.29) | ( 2.73 2.27,3.29) | ( 1.71 1.47,1.98) | ( 0.87 0.71,1.05) | ( 53.44 46.23,61.6) | ( 21.93 18.34,25.85) |
| **Jordan** | ( 0.76 0.46,1.24) | ( 0.46 0.29,0.67) | ( 0.54 0.35,0.79) | ( 0.54 0.35,0.79) | ( 0.78 0.48,1.29) | ( 0.46 0.29,0.67) | ( 22.79 13.86,36.63) | ( 12.96 8.58,18.94) |
| **Kazakhstan** | ( 2.81 1.97,3.82) | ( 1.01 0.68,1.45) | ( 1.18 0.8,1.65) | ( 1.18 0.8,1.65) | ( 2.83 1.99,3.87) | ( 1.03 0.69,1.48) | ( 93.46 66.87,124.54) | ( 31.73 21.59,43.79) |
| **Kuwait** | ( 1.56 1.15,2.04) | ( 0.33 0.23,0.47) | ( 0.41 0.28,0.57) | ( 0.41 0.28,0.57) | ( 1.56 1.15,2.05) | ( 0.32 0.22,0.46) | ( 46.49 35.16,59.72) | ( 8.45 5.78,11.77) |
| **Kyrgyzstan** | ( 1.74 1.24,2.4) | ( 0.76 0.49,1.11) | ( 0.86 0.56,1.25) | ( 0.86 0.56,1.25) | ( 1.76 1.27,2.42) | ( 0.78 0.49,1.14) | ( 59.55 42.54,82.59) | ( 23.09 15.09,33.55) |
| **Lao People's Democratic Republic** | ( 4.4 2.73,6.47) | ( 2.55 1.63,3.86) | ( 2.95 1.9,4.4) | ( 2.95 1.9,4.4) | ( 4.5 2.81,6.67) | ( 2.60 1.66,3.98) | ( 144.52 87.5,212.36) | ( 81.37 51.99,123.22) |
| **Lebanon** | ( 1.52 1.09,2.1) | ( 1.04 0.73,1.42) | ( 1.29 0.92,1.74) | ( 1.29 0.92,1.74) | ( 1.57 1.12,2.15) | ( 1.01 0.71,1.39) | ( 45.37 32.33,62.49) | ( 29.5 20.97,39.62) |
| **Malaysia** | ( 2.31 1.69,3.02) | ( 2.95 2.13,4.01) | ( 3.48 2.52,4.65) | ( 3.48 2.52,4.65) | ( 2.4 1.75,3.14) | ( 2.95 2.12,4.04) | ( 69.56 51.35,90.25) | ( 85.98 61.66,114.41) |
| **Maldives** | ( 3.12 2.15,4.37) | ( 1.71 1.14,2.47) | ( 1.98 1.34,2.86) | ( 1.98 1.34,2.86) | ( 3.28 2.25,4.6) | ( 1.74 1.18,2.52) | ( 92.75 63.85,130.17) | ( 45.64 30.72,65.05) |
| **Mongolia** | ( 17.56 11.4,26.41) | ( 16.61 10.77,24.42) | ( 18.53 12.24,27.4) | ( 18.53 12.24,27.4) | ( 17.97 11.66,27.09) | ( 17.18 11.03,25.21) | ( 571.92 370.05,857.5) | ( 518.05 339.3,773.93) |
| **Myanmar** | ( 1.47 0.68,2.86) | ( 1.21 0.6,2.48) | ( 1.42 0.72,2.91) | ( 1.42 0.72,2.91) | ( 1.51 0.7,2.93) | ( 1.23 0.61,2.50) | ( 48.32 22.23,96.6) | ( 38.32 19.42,78.97) |
| **Nepal** | ( 0.51 0.32,0.77) | ( 0.81 0.48,1.24) | ( 0.92 0.56,1.4) | ( 0.92 0.56,1.4) | ( 0.52 0.33,0.81) | ( 0.84 0.49,1.28) | ( 16.47 10.53,24.99) | ( 25.26 15.19,38.68) |
| **Oman** | ( 1.21 0.71,2.01) | ( 1.23 0.79,1.78) | ( 1.48 0.96,2.14) | ( 1.48 0.96,2.14) | ( 1.23 0.73,2.05) | ( 1.21 0.78,1.75) | ( 37.3 22.31,61.97) | ( 35.47 23.32,50.54) |
| **Pakistan** | ( 0.41 0.29,0.56) | ( 0.5 0.36,0.68) | ( 0.6 0.43,0.82) | ( 0.6 0.43,0.82) | ( 0.42 0.3,0.58) | ( 0.51 0.37,0.70) | ( 13.77 9.98,18.94) | ( 17.16 12.45,23.67) |
| **Palestine** | ( 2.32 1.5,3.49) | ( 1.69 1.19,2.41) | ( 1.91 1.34,2.67) | ( 1.91 1.34,2.67) | ( 2.45 1.58,3.63) | ( 1.74 1.21,2.49) | ( 66.81 44.21,97.65) | ( 47.77 33.71,66.77) |
| **Philippines** | ( 3.27 2.24,4.34) | ( 2.43 1.96,3.03) | ( 2.88 2.32,3.59) | ( 2.88 2.32,3.59) | ( 3.3 2.24,4.42) | ( 2.44 1.97,3.06) | ( 113.33 78.71,145.32) | ( 79.37 63.79,98.77) |
| **Qatar** | ( 3.78 2.45,5.58) | ( 4.17 2.64,6.48) | ( 4.86 3.1,7.43) | ( 4.86 3.1,7.43) | ( 3.98 2.6,5.75) | ( 4.15 2.63,6.43) | ( 105.2 69.33,156.84) | ( 102.58 64.2,156.72) |
| **Republic of Korea** | ( 22.19 15.78,28.39) | ( 11.18 8.91,14.02) | ( 21.97 17.5,27.73) | ( 21.97 17.5,27.73) | ( 21.86 15.57,28.15) | ( 7.91 6.22,10.05) | ( 648.02 462.39,836.33) | ( 213.57 170.61,269.91) |
| **Saudi Arabia** | ( 2.88 1.78,4.55) | ( 1.98 1.28,2.88) | ( 2.2 1.44,3.17) | ( 2.2 1.44,3.17) | ( 2.97 1.82,4.73) | ( 2.03 1.29,2.94) | ( 86.97 53.94,136.22) | ( 53.24 34.59,76.95) |
| **Singapore** | ( 5.77 4.84,6.8) | ( 4.37 3.5,5.29) | ( 8.23 6.63,10.02) | ( 8.23 6.63,10.02) | ( 5.56 4.64,6.54) | ( 3.29 2.62,3.99) | ( 151.66 129.28,175.18) | ( 79.04 64.28,93.31) |
| **Sri Lanka** | ( 0.83 0.61,1.06) | ( 0.53 0.32,0.85) | ( 0.66 0.39,1.05) | ( 0.66 0.39,1.05) | ( 0.86 0.64,1.12) | ( 0.52 0.31,0.82) | ( 25.1 18.92,32.04) | ( 15.41 9.24,24.13) |
| **Syrian Arab Republic** | ( 2.3 1.47,3.44) | ( 1.55 0.95,2.36) | ( 1.79 1.11,2.69) | ( 1.79 1.11,2.69) | ( 2.38 1.51,3.55) | ( 1.57 0.98,2.38) | ( 71.26 46.11,105.57) | ( 44.57 27.65,67.55) |
| **Taiwan (Province of China)** | ( 4.36 3.72,5.08) | ( 5.28 4.24,6.51) | ( 8.24 6.65,10.08) | ( 8.24 6.65,10.08) | ( 4.2 3.57,4.91) | ( 4.48 3.57,5.55) | ( 145.7 126.8,166.15) | ( 135.08 110.93,163.63) |
| **Tajikistan** | ( 1.01 0.6,1.64) | ( 0.72 0.37,1.24) | ( 0.83 0.45,1.38) | ( 0.83 0.45,1.38) | ( 1.03 0.61,1.67) | ( 0.74 0.38,1.30) | ( 34.17 20.53,53.66) | ( 23.04 12.72,38.14) |
| **Thailand** | ( 6.41 4.69,8.69) | ( 4.35 2.97,6.18) | ( 5.7 3.93,8.07) | ( 5.7 3.93,8.07) | ( 6.54 4.8,8.94) | ( 4.13 2.78,5.88) | ( 202.87 151.2,274.89) | ( 133.11 91.85,186.62) |
| **Timor-Leste** | ( 1.41 0.88,2.28) | ( 1.1 0.63,1.98) | ( 1.25 0.71,2.21) | ( 1.25 0.71,2.21) | ( 1.46 0.9,2.34) | ( 1.13 0.65,2.01) | ( 44.52 28,71.08) | ( 33.85 19.33,59.24) |
| **Turkey** | ( 1.39 1.02,1.84) | ( 1.19 0.86,1.62) | ( 1.4 1,1.89) | ( 1.4 1,1.89) | ( 1.45 1.05,1.93) | ( 1.20 0.87,1.65) | ( 41.26 30.91,53.12) | ( 32.95 23.66,44.62) |
| **Turkmenistan** | ( 1.51 1.05,2.04) | ( 1.22 0.76,1.89) | ( 1.43 0.92,2.18) | ( 1.43 0.92,2.18) | ( 1.54 1.06,2.1) | ( 1.23 0.78,1.92) | ( 50.02 35.93,66.78) | ( 40.13 26.02,60.25) |
| **United Arab Emirates** | ( 3.1 1.89,4.67) | ( 3.2 2.08,4.79) | ( 3.55 2.35,5.25) | ( 3.55 2.35,5.25) | ( 3.21 1.98,4.8) | ( 3.31 2.15,5.01) | ( 91.57 58.34,133.47) | ( 88.83 58.64,130.87) |
| **Viet Nam** | ( 6.96 4.53,9.71) | ( 5.25 3.4,7.94) | ( 6.57 4.35,9.92) | ( 6.57 4.35,9.92) | ( 7.07 4.61,9.75) | ( 5.09 3.28,7.62) | ( 224.98 147.4,308.24) | ( 161.33 106.43,246.15) |
| **Yemen** | ( 0.98 0.35,2) | ( 0.59 0.31,1.09) | ( 0.67 0.35,1.23) | ( 0.67 0.35,1.23) | ( 1.01 0.36,2.09) | ( 0.62 0.32,1.13) | ( 31.26 11.6,62.43) | ( 18.3 9.59,33.44) |
