# Supplementary figures and images for: Burden of hepatitis B virus-associated liver cancer in Asia: findings from the global burden of disease study
Source: Front Public Health. 2026 Apr 23;14:1805052. doi: 10.3389/fpubh.2026.1805052 (PMC13149405; doi:10.3389/fpubh.2026.1805052)

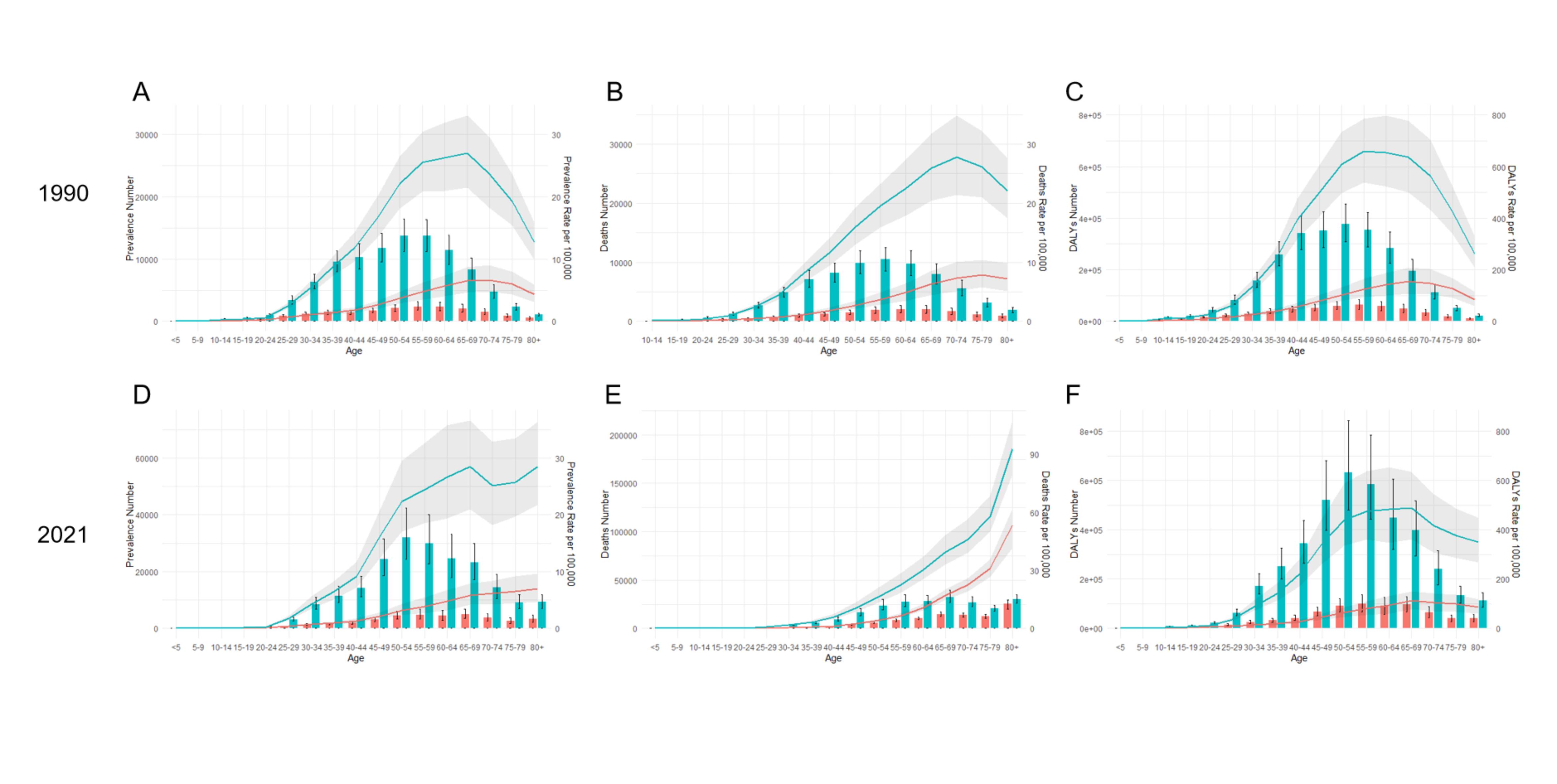

Supplement: Supplementary file 2 [file Image_1.jpeg]

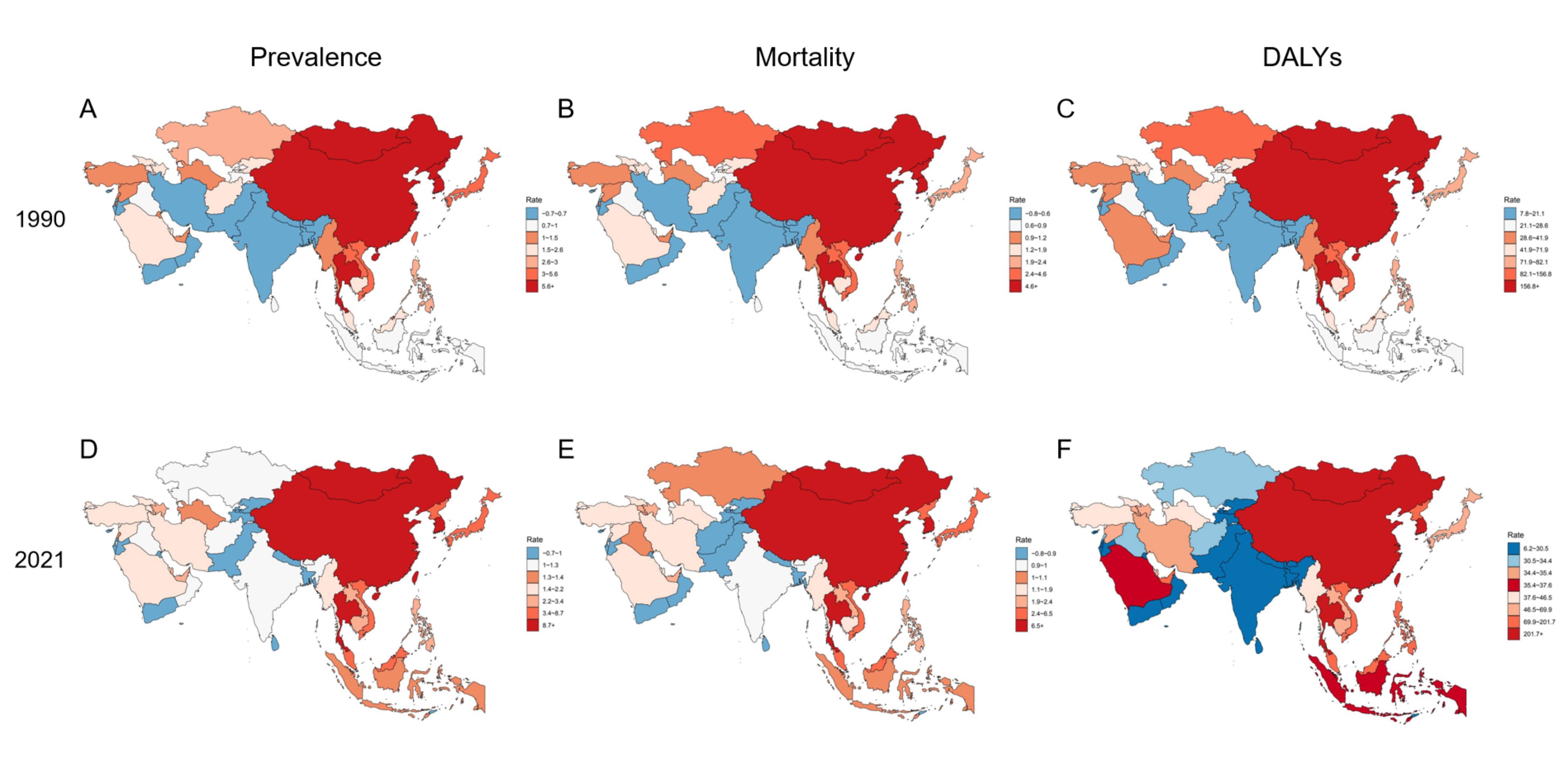

Supplement: Supplementary file 3 [file Image_2.jpeg]

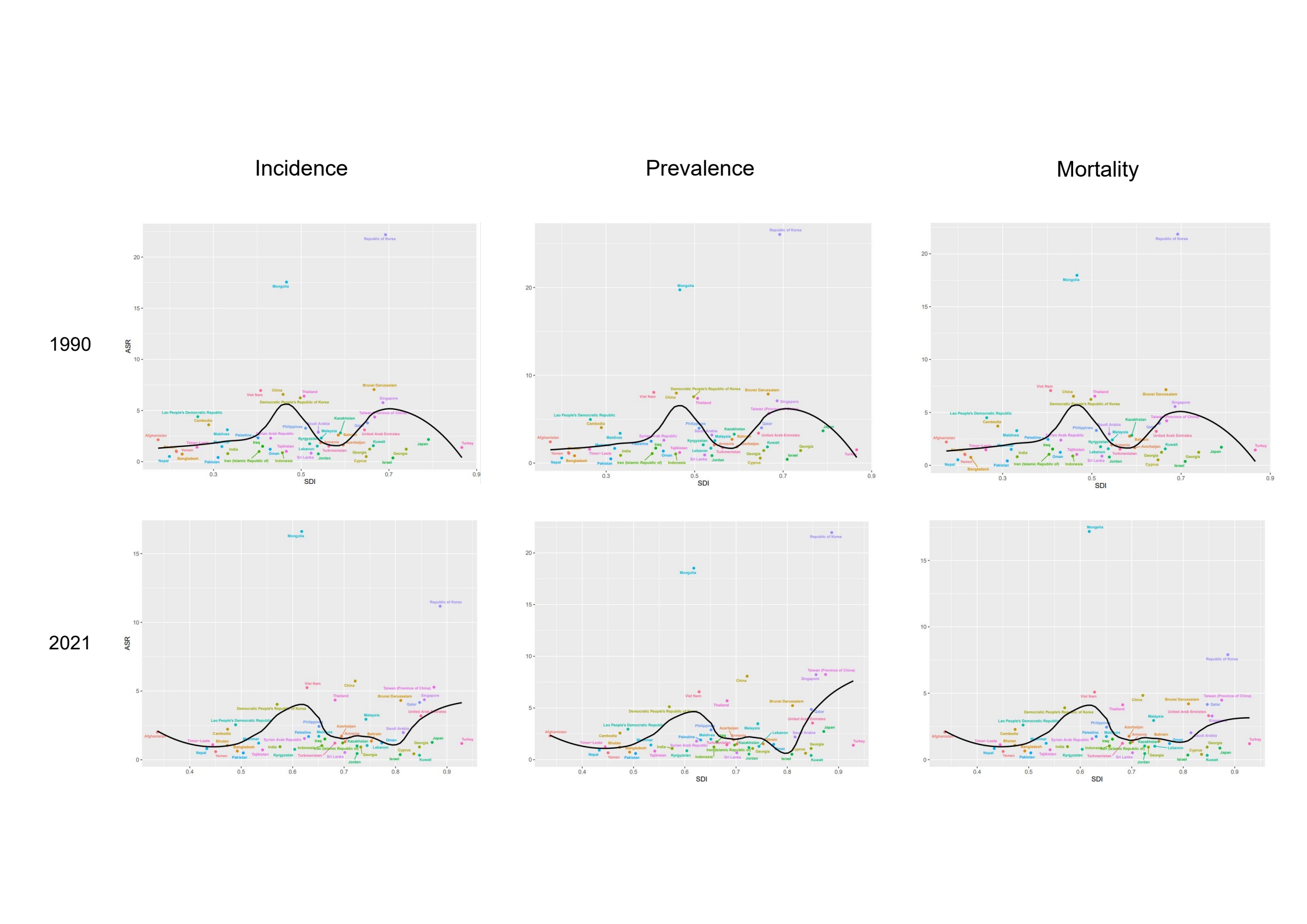

Supplement: Supplementary file 4 [file Image_3.jpeg]
